# Ethnopharmacology and ecosystem applications of woody plant species in the Southern European Alps: a systematic review

**DOI:** 10.3389/fphar.2026.1729802

**Published:** 2026-03-10

**Authors:** Matteo Orlando, Parham Joolaei Ahranjani, Giovanna Ferrentino, Maria Concetta Tenuta, Stefan Zerbe

**Affiliations:** 1 Faculty of Agricultural, Environmental and Food Sciences, Free University of Bozen-Bolzano, Bolzano, Italy; 2 Institute of Geography, University of Hildesheim, Hildesheim, Germany; 3 University of Applied Sciences and Arts, Göttingen, Germany

**Keywords:** alpine plants, cultural identity, ethnopharmacology, medicinal trees and shrubs, phytochemistry

## Abstract

**Introduction:**

Alpine woody plants are deeply embedded in traditional healthcare systems across the Southern European Alps, where multiple organs—including leaves, bark, fruits, buds, and flowers—are used to manage respiratory, musculoskeletal, gastrointestinal, dermatological, metabolic, cardiovascular, and urogenital disorders. This systematic review synthesizes ethnopharmacological uses, evaluates phytochemical and pharmacological evidence, and contextualizes the ecosystem functions of woody plant species native to the European Alps.

**Methods:**

Following PRISMA guidelines, Web of Science, Scopus, and PubMed were systematically searched for peer-reviewed studies published up to May 2025. Data from 281 eligible sources were harmonized, covering 54 woody species (28 trees, 26 shrubs; 25 families). Extracted variables included ecological distribution, harvested organs, phytochemical classes, and experimentally validated bioactivities. Therapeutic indications were mapped using the International Classification of Primary Care (ICPC-2).

**Results:**

Phytochemical profiles were predominantly characterized by phenolic acids, flavonoids, anthocyanins, tannins, terpenoids, alkaloids, and saponins. These compounds underpin convergent anti-inflammatory, antimicrobial, antioxidant, antidiabetic, vasoprotective, and cytotoxic activities demonstrated in vitro and in vivo. Beyond medicinal relevance, the same species contribute to soil stabilization, hydrological regulation, carbon sequestration, and the preservation of biocultural landscapes. However, climate-driven range shifts, land-use intensification, commercial overharvesting, and limited pharmacokinetic and toxicological data constrain both sustainability and clinical translation.

**Discussion:**

High-priority taxa and critical knowledge gaps were identified. An integrated research framework is proposed, combining standardized green extraction technologies, high-resolution metabolomics, longitudinal ecological monitoring, participatory documentation of traditional knowledge, and equitable benefit-sharing mechanisms. Such integration is necessary to advance evidence-based phytotherapy while ensuring sustainable stewardship of Alpine woody ecosystems.

## 1 Introduction

The European Alps, spanning across eight countries and covering approximately 200,000 km^2^, represent a globally significant biodiversity hotspot and a dynamic socio-ecological system where natural and cultural diversity co-evolved (Balestrini and Tagliaferri, 2001). This mountain region is characterized by an exceptionally rich flora, with over 4,000 vascular plant taxa documented (Wilhalm et al., 2014), including a diverse array of woody species that are both, ecologically and culturally indispensable (Ambrosi et al., 2003). The ecological functions and services of these trees and shrubs are multifaceted. They regulate critical ecosystem processes such as soil stabilization, hydrological buffering, nutrient cycling, carbon sequestration, and microclimatic modulation (Scalenghe et al., 2002; Vitalini et al., 2013). Moreover, they shape the physiognomy and functionality of Alpine forest ecosystems, contribute to habitat heterogeneity, and support biodiversity at multiple trophic levels (Hood and Williams, 2001; Rainer et al., 2016; Sulimanec et al., 2023).

Beyond their ecological significance, woody plants in the European Alps hold deep ethnobotanical and ethnopharmacological values (Danna et al., 2022). For centuries, Alpine communities have developed and transmitted extensive local knowledge concerning the medicinal and functional uses of native woody species (Alrhmoun et al., 2025; Danna et al., 2022). Historical records and field surveys indicate that nearly all morphological parts of woody plants, comprising bark, leaves, buds, flowers, fruits, cones, seeds, and roots, have been harnessed for treating a wide range of diseases, including respiratory conditions, rheumatic diseases, skin disorders, gastrointestinal problems, infections, and metabolic imbalances (Stucki et al., 2019). Species such as *Abies alba* Mill., *Betula pendula Roth*, *Juniperus communis* L., *Larix decidua* Mill., *Pinus mugo Turra*, and *Vaccinium myrtillus* L. feature prominently in Alpine pharmacopoeias, with preparations ranging from decoctions, infusions, tinctures, balms, and syrups to fermented extracts and distilled essential oils (Venditti et al., 2013; Pop et al., 2015; Baldan et al., 2017; Ferreira et al., 2017; Ancuceanu et al., 2023; Gargiulo et al., 2024). The phytochemical richness of these woody species underpins their therapeutic efficacy (Burylo et al., 2012; Turnbull et al., 2013; Fernández-Marín et al., 2018).

Numerous studies have identified and quantified a wide spectrum of bioactive secondary metabolites in Alpine woody plants, including polyphenols (e.g., flavonoids, phenolic acids, tannins), terpenoids (e.g., monoterpenes, sesquiterpenes, diterpenes), alkaloids, lignans, and coumarins (Zhou et al., 2022; Sulimanec et al., 2023; Ahranjani et al., 2025). These compounds exhibit pharmacological activities such as anti-inflammatory, antimicrobial, antiviral, antioxidant, analgesic, and antispasmodic effects, some of which have been validated via *in vitro* assays, *in vivo* models, and, in rare cases, clinical trials (Cui et al., 2016; Sulimanec et al., 2023). For example, essential oils from *Picea abies* (L.) H. Karst. and *Pinus sylvestris* L. show notable antibacterial and anti-inflammatory properties, while berry extracts of *Vaccinium vitis-idaea* and *Vaccinium myrtillus* are rich in anthocyanins with antioxidant and antidiabetic potential (Salem et al., 2016; Macovei et al., 2023; Sandulovici et al., 2024). Importantly, the phytochemical profiles of these species are influenced by environmental variables such as altitude, soil composition, phenological stage, and harvesting time, all of which can modulate the concentration and bioavailability of active constituents (Streit et al., 2014).

Despite this rich ethnopharmacological heritage (Mattalia et al., 2023; Gerner et al., 2025) and emerging phytochemical evidence, comprehensive approaches that bridge ecological, pharmacological, and socio-cultural dimensions of Alpine woody plants remain scarce. The fragmentation of knowledge across linguistic, disciplinary, and geographical boundaries has limited the development of integrative frameworks that can consider both, the conservation of plant diversity and their sustainable use. Simultaneously, climate change is accelerating ecological transformations in Alpine regions, with rising temperatures, altered precipitation patterns, and shifting disturbance regimes (e.g., wildfires, pest outbreaks, storm damage) that directly affect the distribution, phenology, and health of woody plant populations (Lamentowicz et al., 2010; Kotlarski et al., 2023). These changes may also impact on the quality and availability of the bioactive compounds obtained from them, complicating sustainable harvesting and cultivation strategies (Piccand et al., 2019; Jarque-Bascuñana et al., 2022). Furthermore, woody plants contribute significantly to ecosystem services that intersect human health and wellbeing. Beyond their role in regulating ecosystem functions, many species offer provisioning services (e.g., fuelwood, timber, wild foods, and medicinal raw materials), cultural services (e.g., spiritual symbolism, identity, and heritage landscapes), and supporting services such as pollination and soil formation (Jia et al., 2022; Joolaei Ahranjani et al., 2025a). Several species are keystone or flagship taxa whose presence and management shape broader ecological assemblages and socio-ecological practices (Petelka et al., 2022). The recognition of such multifunctionality is critical for promoting sustainable landscape management approaches (Zerbe, 2022) that integrate pharmacological valorization with ecological stewardship.

Natural products constitute a major reservoir of bioactive molecules with long-standing relevance in traditional medicine and modern pharmacology. Their therapeutic potential spans antioxidant, anti-inflammatory, antimicrobial, cytotoxic, immunomodulatory and metabolic activities, supporting their use as templates for drug discovery and preventive healthcare strategies (Ebadi and Selamoglu, 2025; Salehi et al., 2019). Recent advances in phytochemistry and molecular pharmacology have demonstrated how plant-derived compounds-including phenolics, alkaloids, terpenoids, and ribosome-inactivating proteins-exert targeted biological effects relevant to chronic diseases, cancer, infectious disorders and metabolic dysfunctions (Selamoglu et al., 2024; Ebadi et al., 2025b; Ebadi et al., 2025a; Selamoğlu et al., 2025). In the present review, we build on this global body of methodological and mechanistic work but deliberately restrict our pharmacological evidence base to the 54 woody species that are native or characteristic of the European Alps and have documented medicinal use in this region. Preclinical and clinical studies conducted outside the Alps are only considered when they directly investigate one of these 54 taxa, ensuring that experimental data are tightly aligned with Alpine ethnopharmacology rather than representing a random assemblage of pharmacological reports.

Given the increasing scientific interest in natural products and plant-derived therapeutics, particularly in the context of antimicrobial resistance and chronic disease management (Danna et al., 2022; Joolaei Ahranjani et al., 2025b), Alpine woody plants therefore represent an underexplored but clearly delimited reservoir of bioactive compounds with pharmaceutical potential. However, research efforts within this set of taxa have often been uneven, focusing on a limited subset of well-known species or relying on traditional usage claims without adequate phytochemical or pharmacological validation. Methodological discrepancies, including variable extraction procedures, inconsistent bioassay protocols and incomplete taxonomic documentation, further complicate comparative assessments and evidence synthesis. Moreover, the lack of a systematic approach to reviewing ethnopharmacological and ecological data has hindered the formulation of coherent research agendas and policy recommendations.

However, research efforts have often been uneven in light of these challenges and opportunities, the present systematic review aims to consolidate and critically evaluate the state of knowledge on the ethnopharmacological uses and ecosystem applications of woody plant species native to the European Alps. Covering peer-reviewed literature from the earliest available records until May 2025, this review integrates historical and contemporary sources to provide a comprehensive synthesis of (i) medicinal uses and ethnopharmacological traditions, (ii) phytochemical composition and pharmacological properties, and (iii) ecological roles and ecosystem service contributions of Alpine woody species. By identifying high-potential taxa, elucidating phytochemical-efficacy relationships, and highlighting conservation and research gaps, this study aims to provide a reference for pharmacognosy, mountain ecology, ethnobotany, and sustainable bioprospecting in Alpine contexts.

## 2 Methodology

### 2.1 Literature search strategy

A systematic and comprehensive literature review was conducted to compile and critically analyze available scientific knowledge on the ethnopharmacological relevance and ecosystem applications of woody plant species native to the European Alps. The review was designed in accordance with the Preferred Reporting Items for Systematic Reviews and Meta-Analyses (PRISMA) guidelines to ensure methodological transparency, reproducibility, and quality (Moher et al., 2009). Three major scientific databases, comprising the Web of Science (Clarivate Analytics), Scopus (Elsevier), and PubMed were systematically queried using botanical, regional nomenclature, and pharmacological terminology (Supplementary Material).

**FIGURE 1:**
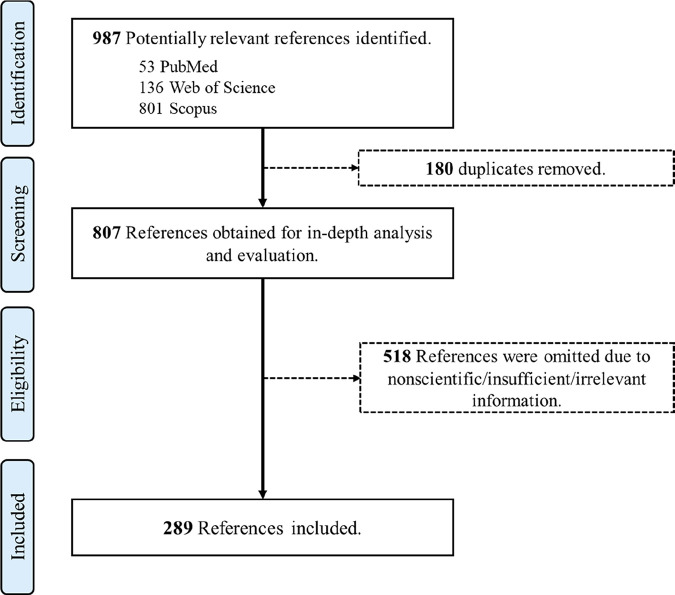
PRISMA flow diagram illustrating the study selection process for the current systematic review, including identification, screening, eligibility, and inclusion stages.

The core of the search strategy consisted of entering the scientific names of each target woody species into the “All Fields” option of each database, combined with relevant keywords such as “medicinal use”, “traditional medicine”, “Alps”, “ethnobotany”, “ethnopharmacology”, “ecosystem services”, and “therapeutic potential”, etc. Boolean operators and truncation were used to enhance sensitivity. The search included peer-reviewed literature and selected high-quality literature published from the earliest available records until May 2025, to ensure exhaustive historical and contemporary coverage (Figure 1).

### 2.2 Taxonomic scope and inclusion criteria

The scope of the review was established based on an initial list of more than 200 plant species documented in traditional medicinal use within the Alpine region (Petelka et al., 2020). From this broader list, all woody species-defined here as trees and shrubs exhibiting persistent lignified stems and branches-were retained for focused analysis. This resulted in a final dataset of 54 woody species, comprising 28 trees and 26 shrubs. Species classification into these growth forms was based on a growth height threshold, where shrubs were defined as typically <5 m in mature height and trees >5 m, in line with standard ecological criteria (Prosser et al., 2019). The taxonomic nomenclature of all species was harmonized according to the International Plant Names Index (IPNI, 2020). Identification of taxa below the infraspecific level (e.g., subspecies, varieties, autonyms) was verified using IPNI and regional floras, following accepted nomenclatural rules to ensure correct authorship attribution and taxonomic validity. Corresponding plant family designations were cross-referenced with the regional biodiversity portal to ensure consistency with local floristic databases (Wilhalm et al., 2014). Nativity and biogeographical distribution within the Southern European Alps were verified using regional floras and biodiversity databases (e.g., Wilhalm et al., 2014; Prosser et al., 2019), allowing us to distinguish strictly Alpine or sub-Alpine taxa from more widespread European-Eurasian species that occur in the study area. To be included in the review, studies had to meet the following eligibility criteria: (i) provide empirical data or verifiable ethnobotanical records of medicinal use for at least one of the selected 54 woody species; (ii) report on the identification or characterization of phytochemicals; (iii) document pharmacological or therapeutic activities validated through traditional knowledge, *in vitro*/*in vivo* experiments, or clinical observations; (iv) include information on ecological traits relevant to their natural habitat, occurrence, and harvest potential within the Alpine region; (v) address ecosystem services or functional roles of the species in Alpine landscapes. Authorship of scientific names follows IPNI standards, and full authorship is provided at the first mention of each species in the main text. Excluded from the analysis were non-peer-reviewed opinion papers, conference abstracts without full texts, studies lacking plant identification at the species level, and publications solely addressing ornamental, timber, or non-medicinal uses.

### 2.3 Screening and selection process

The literature screening process was conducted in three stages. In the first phase, search results were consolidated using a reference management system to remove duplicates and facilitate citation tracking. Titles and abstracts were then screened for relevance to the study’s scope, guided by the pre-defined inclusion criteria. In the second phase, full-text articles of potentially eligible studies were retrieved and assessed in detail for compliance with methodological standards and thematic relevance. Screening and selection were independently performed by two reviewers with expertise in ethnobotany and phytochemistry. Discrepancies were resolved through discussion, and where necessary, adjudicated by a third reviewer.

### 2.4 Data extraction and synthesis

A structured data extraction template was developed to ensure uniformity and completeness in the synthesis of findings. The following categories of information were extracted for each species: (i) biological and ecological parameters, including growth form, average plant height (in m), altitudinal distribution range (categorized into planar, colline, montane, subalpine, alpine, and nival belts), and flowering period based on phenological data (Prosser et al., 2019); (ii) harvest characteristics, such as plant parts used (e.g., leaves, bark, fruits, cones, buds, flowers), the state of plant material (fresh or dried), and timing of collection relative to the balsamic period being the phase of maximal phytochemical expression, often coinciding with anthesis (Bruni, 1999; Sannia and Careddu, 2010; Selamoglu et al., 2023); (iii) phytochemical data, organized by compound class (e.g., flavonoids, terpenoids, alkaloids, phenolic acids, saponins, lignans, tannins, carotenoids, coumarins, amino acids, and vitamins), including identification techniques and reported concentrations where available; (iv) pharmacological and therapeutic information, encompassing biological activities (e.g., antimicrobial, antioxidant, anti-inflammatory, cytotoxic, antidiabetic) and associated health uses, documented through traditional knowledge, experimental assays, or pharmacodynamic studies. To avoid an unsystematic mixture of evidence, pharmacological and phytochemical data were extracted only for the 54 woody species identified as medicinal in the Alpine ethnobotanical screening; studies dealing exclusively with non-Alpine or non-woody taxa were not included.

To systematically categorize the health indications of each species, the International Classification of Primary Care (ICPC-2) was applied (World Health Organization, 2009). The ICPC-2 chapters used for this classification are listed (Table 1). This framework enables a standardized mapping of therapeutic indications to clinical systems, facilitating cross-study comparability and integration with modern primary care taxonomies.

**TABLE 1:** The chapters of International Classification of Primary Care (ICPC) for the second edition of ICPC-2.

ICPC-2 chapters
A – General and unspecified
B – Blood, Blood forming organs and immune mechanism
D – Digestive
F – Eye
H – Ear
K – Cardiovascular
L – Musculoskeletal
N – Neurological
P – Psychological
R – Respiratory
S – Skin
T – Endocrine, Metabolic and Nutritional
U – Urological
X – Pregnancy, Childbearing, Family Planning
Y – Female genital
Z – Male genital
W – Social problems

In the final synthesis, species profiles were constructed based on the integrated ecological data, phytochemical properties, and documented therapeutic applications. These profiles were analyzed qualitatively and quantitatively to identify patterns of bioactivity, multipurpose usage, and conservation relevance. Cross-referencing of bibliographies and targeted backward citation tracking were also employed to identify any relevant studies not captured in the initial database searches, enhancing the completeness of the review. The summarized table is present to show the key variables used in species profiling, including growth form, altitudinal distribution, flowering season, harvested organs, tissue state, and classified phytochemical groups (Table 2). These criteria provided the basis for standardized comparison across taxa, and ensured consistency in evaluating pharmacological relevance within ecological context.

**TABLE 2:** Biological parameters of woody plant species considered in our study and information on usage parameters (e.g., plant parts).

Parameter	Explanation	Source
Biological and ecological parameters		
Life form	According to growth height with shrubs <5 m and trees >5 m	Wilhalm et al. (2014)
Plant height	Mean of plant height in meter	Prosser et al. (2019)
Flowering season	Months of the vegetation season in which the species is flowering	
Occurrence above sea level	Altitudinal mountain belts where the species generally occur with planar (lowest altitudinal range), colline, montane, sub-alpine, alpine, nival (highest range)	

## 3 Results

### 3.1 Floristic and taxonomic diversity

The selected dataset comprises 54 woody plant species with documented medicinal or ethnopharmacological use in the European Alps, encompassing 28 tree species and 26 shrub species. These taxa are distributed across 25 plant families, reflecting a broad floristic representation of Alpine woody vegetation with both angiosperms and gymnosperms included. Among the shrubs, the families with the highest species richness are Rosaceae (7 species) and Ericaceae (5 species), together accounting for nearly half of the medicinal shrubs considered. Additional families represented by at least two shrub species include Caprifoliaceae, Rhamnaceae, and Fabaceae, while several others, such as Cornaceae and Thymelaeaceae, are represented by single medicinal taxa. In the tree group, the highest number of medicinally used species belong to Pinaceae (6 species), followed by Salicaceae (5 species), Fagaceae (4 species), and Rosaceae (4 species). These families represent ecologically dominant lineages in Alpine forests and subalpine shrublands (Körner, 1989; Prosser et al., 2019), and their pharmacological relevance is consistent with their structural and functional prominence in these ecosystems.Comparative analysis reveals that shrubs are distributed across a greater number of plant families (14) than trees (11 families), indicating a higher taxonomic diversity among medicinal shrubs. The complete taxonomic enumeration, including species names, family affiliations, growth forms, and associated medicinal use references is present where 54 medicinal woody species are reviewed, with taxonomic affiliation, flowering phenology, growth height, and altitudinal distribution across Alpine mountain belts (Table 3). The distribution is reported from the area where the species are most present to the area where they are less present. In addition, (Table 3), reports the nativity status and geographic range of each taxon (endemic or characteristic of the Southern European Alps versus broadly distributed European or Eurasian species), together with the principal floristic sources used to confirm their occurrence in the region.

**TABLE 3:** Studied woody plant species, differentiated in shrubs and trees, with plant family, flowering period, growth height (m), and distribution (occurrence as N: nival; A: alpine; S: subalpine; M: mountane; C: colline; P: planar) (Prosser et al., 2019)), including a column reporting nativity and geographic origin within the Southern European Alps.

No.	Scientific name	Family	Flowering period	Height (m)	Distribution	General nativity
*Shrubs*						
1	*Arctostaphylos uva-ursi* (L.) Spreng	*Ericaceae*	4–7	0.2–1	A; S; M; C	Alpine–Subalpine native
2	*Berberis vulgaris* L	*Berberidaceae*	4–6	0.5–2.5	S; M; C; P	Native but widespread in Europe
3	*Calluna vulgaris* (L.) Hull	*Ericaceae*	7–11	0.1–0.9	A; S; M	Native to Southern European Alps
4	*Corylus avellana* L	*Betulaceae*	2–4	1–5	M; C	Native but widespread in Europe
5	*Crataegus monogyna* Jacq	*Rosaceae*	4–6	1–6	M; C; P	Widespread Eurasian species (native)
6	*Erica carnea* subsp. *carnea* L	*Ericaceae*	6–10	0.15–0.3	A; S; M; C	Native to Southern Alps (Subalpine specialty)
7	*Genista tinctoria* L	*Fabaceae*	5–8	0.3–0.5	M; C; P	Native but widespread in Europe
8	*Hedera helix* L	*Araliaceae*	8–11	1–15	M; C; P	Native but widespread in Europe
9	*Hippophae rhamnoides* subsp. *fluviatilis* Soest	*Elaeagnaceae*	3–5	0.5–5	M; C; P	Native riparian shrub of Southern Alps
10	*Humulus lupulus* L	*Cannabaceae*	5–8	3–6	M; C; P	Widespread Eurasian species (native)
11	*Ilex aquifolium* Lour	*Aquifoliaceae*	4–6	1–8	M; C	Native but widespread in Europe
12	*Myricaria germanica* (L.) Desv	*Tamaricaceae*	5–8	1–3	S; M	Alpine riverine native (specialist)
13	*Ononis spinosa* Hasselq	*Fabaceae*	5–9	0.2–0.6	M; C; P	Native but widespread in Europe
14	*Ribes petraeum* Wulfen	*Grossulariaceae*	4–6	1–2	A; S	Alpine–Subalpine native
15	*Rosa canina* Sol. ex Bab	*Rosaceae*	5–7	0.3–2	S; M; C; P	Native but widespread in Europe
16	*Rosa corymbifera* Borkh	*Rosaceae*	5–7	0.3–2	S; M; C; P	Native but widespread in Europe
17	*Rosa* x *damascena* Mill	*Rosaceae*	5–9	1–2	A; S; M; C; P	Historically naturalized; cultivated and present in Alps
18	*Rosa montana* Chaix ex Vill	*Rosaceae*	6–7	1–3	M	Native to the Southern Alps
19	*Rosa pendulina* L	*Rosaceae*	6–7	1–2	A; S; M	Alpine–Subalpine native
20	*Rubus idaeus* L. subsp*. Idaeus*	*Rosaceae*	6–7	1–2	S; M	Native but widespread in Europe
21	*Ruscus aculeatus* L	*Asparagaceae*	3–5	0.3–1	C; P	Native to Southern Europe (incl. Southern Alps)
22	*Sambucus ebulus* L	*Adoxaceae*	6–8	0.6–2	S; M; C	Native but widespread in Europe
23	*Sambucus racemosa* L	*Adoxaceae*	4–5	1–4	S; M	Native to European montane/alpine regions
24	*Vaccinium myrtillus* Cham. & Schltdl	*Ericaceae*	4–7	0.1–0.5	N; A; S; M	Alpine–Subalpine native
25	*Vaccinium vitis-idaea* W.D.J. Koch	*Ericaceae*	5–7	0.05–0.3	A; S; M	Alpine–Subalpine native
26	*Viscum album* Webb	*Santalaceae*	3–5	0.2–0.5	M; C; P	Native but widespread in Europe
*Trees*						
1	*Abies alba* Mill	*Pinaceae*	5–6	20–50	M	Native to the Southern European Alps
2	*Aesculus hippocastanum* L	*Hippocastanaceae*	4–5	8–25	M; C; P	Native to the Balkans; naturalized in Alps
3	*Betula pendula* Roth	*Betulaceae*	4–5	10–30	S	Native but widespread in Europe
4	*Castanea sativa* Mill	*Fagaceae*	5–7	5–35	M; C	Native to Southern Europe; naturalized in Alps
5	*Fagus sylvatica* L	*Fagaceae*	4–5	25–40	M	Native to the Southern European Alps
6	*Fraxinus excelsior* L	*Oleaceae*	3–5	8–40	M; C	Native but widespread in Europe
7	*Fraxinus ornus* L	*Oleaceae*	4–6	1–10	M; C; P	Native to Southern Europe including the Alps
8	*Juniperus communis* Thunb	*Cupressaceae*	3–5	1–3	M; C	Native but widespread in Europe
9	*Larix decidua* Mill	*Pinaceae*	4–6	20–50	S; M	Alpine endemic species
10	*Picea abies* (L.) H.Karst	*Pinaceae*	4–6	20–50	A; S; M; C; P	Native to Central and Eastern Alps
11	*Pinus cembra* Thunb	*Pinaceae*	6–8	10–25	A; S; M	Alpine–Subalpine native (high-elevation specialist)
12	*Pinus mugo* Turra	*Pinaceae*	6–7	0.5–5	A; S; M; C	Alpine endemic species
13	*Pinus sylvestris* Baumg	*Pinaceae*	5–6	5–30	M; C; P	Native but widespread in Eurasia
14	*Populus tremula* L	*Salicaceae*	3–5	5–20	S; M; C; P	Native but widespread in Europe
15	*Prunus avium* (L.) L	*Rosaceae*	4–5	0.5–25	M; C; P	Native but widespread in Europe
16	*Prunus spinosa* Walter	*Rosaceae*	4–5	0,5–3	M; C; P	Native but widespread in Europe
17	*Pyrus pyraster* (L.) Burgsd	*Rosaceae*	4–5	5–20	M; C; P	Native to the Southern Alps
18	*Quercus petraea* (Matt.) Liebl	*Fagaceae*	4–5	10–30	M; C	Native but widespread in Europe
19	*Quercus pubescens* Brot	*Fagaceae*	4–5	10–20	M; C	Native to Southern Europe including the Alps
20	*Quercus robur* Asso	*Fabaceae*	4–5	10–40	C	Native but widespread in Europe
21	*Salix alba* Kern	*Salicaceae*	3–5	3–25	M; C; P	Native but widespread in Europe
22	*Salix caprea* Boiss. & Buhse	*Salicaceae*	3–5	1–10	S; M; C	Native but widespread in Europe
23	*Salix pentandra* Kern	*Salicaceae*	5–7	3–12	S; M	Native to boreal/Eurasian regions; present in Alps
24	*Salix purpurea* L	*Salicaceae*	3–5	1–6	S; M; C; P	Native but widespread in Europe
25	*Sambucus nigra* Marshall	*Adoxaceae*	4–7	2–7	M; C; P	Native but widespread in Europe
26	*Sorbus aucuparia* Poir	*Rosaceae*	5–6	1–15	S; M; C	Native to montane–subalpine Alps
27	*Tilia cordata* Mill	*Malvaceae*	6–7	5–30	M; C; P	Native but widespread in Europe
28	*Tilia platyphyllos* Scop	*Malvaceae*	5–6	5–30	M; C	Native but widespread in Europe

### 3.2 Ethnobotanical uses and organ-specific applications

The most frequently used plant organs are leaves, bark, fruits, buds, and flowers, followed by seeds, roots, cones, and trunk tissues. These parts are typically harvested in fresh state and used as they are collected or in dried form depending on the intended preparation and pharmacological efficacy. Leaves and bark were the most widely documented medicinal sources across both trees and shrubs. Leaves of species such as *B. pendula, Rubus fruticosus*, *Salix caprea*, and *V. myrtillus* are used for their anti-inflammatory, diuretic, and antimicrobial properties, often in the form of infusions and decoctions (Moohammadnor et al., 2010; Pellissier, 2013; Pop et al., 2015). Bark extracts, particularly from species in Salicaceae (e.g., *Salix daphnoides, S. pentandra,* and *S. purpurea*), are rich in salicylates and have long been used for treating fever, pain, and rheumatic conditions. Thanks to the traditional use of these barks, it was discovered that salicylates have beneficial effects. For this reason, today these species are taken as active ingredients in pharmaceutical products.

Fruits constitute another pharmacologically relevant organ, especially among taxa in the Rosaceae and Ericaceae families, such as *Rosa canina* L., *Sorbus aucuparia* L., *V. myrtillus* L., and *V. vitis-idaea* L. These fruits are traditionally consumed as teas, syrups, or preserves, and are valued for their high content of anthocyanins, ascorbic acid, and other antioxidants with potential antidiabetic, cardioprotective, and immunostimulatory effects (Vilkickyte et al., 2022). Buds are particularly prized in gemmotherapy and are recognized for their high concentrations of meristematic-derived secondary metabolites (Ryabov et al., 2021; Sandulovici et al., 2024). In both broad-leaved and coniferous species-including *Fraxinus excelsior* L., *L. decidua* Mill., *P. mugo Turra*, *Quercus robur* L., and *S. caprea* L.-bud extracts are employed for inflammatory conditions, lymphatic drainage, and metabolic regulation (Table 4). The pharmacological potency of buds is often associated with their balsamic period, during which the biosynthesis of phytochemicals reaches its peak. This period generally coincides with the onset of anthesis, typically occurring between May and July, although certain taxa (e.g., *Corylus avellana, Erica carnea*) flower earlier or later in the season, extending the potential harvest window.

**TABLE 4:** Documented medicinal uses of woody plant species analysed, including organs used, harvested state, ethnomedicinal indications, culinary roles, and modes of ethnomedicinal preparation.

No.	Scientific name	Organs used	State of collected matter	Ethnomedicinal uses	Culinary uses	Preparations	References
Shrubs							
1	*Arctostaphylos uva-ursi*	Leaf	Dry	Urogenital tract infection (cystitis, prostatitis, uropathogenic bacteria)	Food industry as natural antioxidant agent and food packaging	-	de Arriba et al. (2013), Mohd Azman et al. (2016), Wrona et al. (2019)
2	*Berberis vulgaris*	Bark Fruit Leaf Root Shoot	Dry	Fever Cough, dysentery diarrhea skin disease eye/ear/mouth infections	Fruit industry Juice extraction and carbonated drinks Food cooked Sauce, jelly, jam, marmalade industries Food additives as flavor, garnish foods, sapors and nature-based color	Decoction Infusion Extract	Imenshahidi and Hosseinzadeh (2016), Rahimi-Madiseh et al. (2017)
3	*Calluna vulgaris*	Flower Leaf Shoot	Dry -Fresh	Skin damages produced by sun radiation, skin burns, urinary tract pathogens, depression, gout, ulcer, gastritis	Pharmaceutical uses Healther honey from flower Animal pastoral resource	Infusions	Dezmirean et al. (2015), Bekkai et al. (2022), Cucu et al. (2022)
4	*Corylus avellana*	Bark Bud Fruit Leaf Male Flower Seed	Dry - Fresh	Cardiovascular diseases, relaxing blood vessels pressure, reducing cholesterol and diabetes, mental exhausting, anaemia, varicose veins	Food industry for snacks, desserts, cream Oil food industry to product hazelnut oil to increase the shelf-life of foods	Oil extraction by pressure	Cappelli et al. (2018), Cabo et al. (2021), Çelik et al. (2023), Zhao et al. (2023)
5	*Crataegus monogyna*	Leaf Flower Fruit	Dry - Fresh	cardiovascular diseases atherosclerosis colds/bronchiti diarrhea	Food industry as jellies, jams and syrups Food beverage as liquors Young flowers ans leaves in salads Fruits are eaten raw	Fruit drying time at 60 °C for 16 h Food dishes and raw materials Infusions	Barros et al. (2011), Jarzycka et al. (2013), Alirezalu et al. (2018), Moldovan et al. (2021)
6	*Erica carnea* subsp. *carnea*	Leaf Flower	Dry	Urinary tract infections, cystitis, kidney stones, prostate disorders, uric acid elimination, wound, skin disorders	-	-	Pavlović et al. (2013), Veličković et al. (2017)
7	*Genista tinctoria*	Flower Leaf Root Seed Stem	Dry	Gout Rheumatism Dropsy Thyroid disorders Menopausal symptoms Diuretic uses	Only for medicinal properties	Infusions Decoction Ointment	Rigano et al. (2010), Sharifi-Rad et al. (2021)
8	*Hedera helix*	Fruit Leaf Stem	Dry	Asthma Bronchitis Cough Catarrh	-	-	Delmas et al. (2000), Hong et al. (2015), Akhlaghi et al. (2022), Gavrila et al. (2022), Osama et al. (2023), Qabaha et al. (2023)
9	*Hippophae rhamnoides* subsp. *fluviatilis*	Branch Fruit Leaf Root Seed	Dry - Fresh	Cough Asthma, skin healing wound healing liver support	Ferment Fruits as fresh food Fruit wine Jam Juice Oil Yogurt	-	Buyukokuroglu and Gulcin (2009), Suryakumar and Gupta (2011), Kong et al. (2020), Żuchowski (2023), Ma et al. (2024)
10	*Humulus lupulus*	Flower Fruit Leaf Shoot	Dry - Fresh	Insomnia, nervous tension, digestive disorders menopausal discomfort	Vegetables	-	Nuutinen (2018), Lin et al. (2019), Yan et al. (2021), Bektur et al. (2022)
11	*Ilex aquifolium*	Leaf	Dry	Brain stimulation Urinary infections, digestive issues	-	-	Paluch et al. (2021), Pachura et al. (2022), 2024
12	*Myricaria germanica*	Bark Leaf	Dry	Jaundice Chronic bronchitis Cough, rheumatoid arthritis	-	Infusions Extracts	Nawwar et al. (2013), Liu et al. (2023), Yang et al. (2024)
13	*Ononis spinosa*	Flower Leaf Root	Dry	Diuretic Kidney stones, urinary infections, rheumatism	-	Infusions	Yõlmaz et al. (2006), Benedec et al. (2012), Addotey et al. (2018), Abbas and Jaffal (2019)
14	*Ribes petraeum*	Fruit Leaf	Dry - Fresh	Wound healing, digestive disorders, respiratory problems, arthritis	Food industry as cake, jam, juice, ice - cream, preserves, fermented drinks and liquors Natural dyes (food, hair and textile dyeing)	Ointments Infusions	Šikšnianas et al. (2013), Kendir et al. (2019), Sun et al. (2021)
15	*Rosa canina*	Flower Fruit Leaf Root Seed Stem	Dry - Fresh	Infections Fever GI disorders, skin issues throat problems	Food industry as jam, yogurts, probiotic drinks, snacks, teas, flour and soups	Decoctions Infusions Oil Powder	Ayati et al. (2021), Băbălău-Fuss et al. (2021), Wang et al. (2022b), Demirci Kayiran et al. (2023), Negrean et al. (2023), Manzione et al. (2024), Saini et al. (2024)
16	*Rosa corymbifera*	Fruit Seed	Dry	Cardiovascular Arthritis, wound healing hypertension	Food industry as jam, marmalade, fruit juice, dried fruit Food supplement industry	-	Kayahan et al. (2023), Kunc et al. (2023b)
17	*Rosa x damascena*	Flower	Dry - Fresh	chest pain, digestive problems, cough, depression	Food industry as aromatic, yoghurt, bread, drinks Essential oil and water used in perfumery	Decoctions Infusions Oil Powder	Boskabady et al. (2011), Nayebi et al. (2017), Osama and Ikram (2018), Beheshti et al. (2021), Ramdan et al. (2021), Yaghoobi et al. (2022)
18	*Rosa montana*	Fruit Seed	Dry	Heart diseases, vascular diseases, diabetes	-	-	Yilmaz et al. (2011), Hatipoglu and Ak (2023)
19	*Rosa pendulina*	Flower Fruit Seed	Dry - Fresh	Immunostimulant, arthritis, fever, skin problems	-	-	Karczmarz et al. (2019), Kunc et al. (2022), Kunc et al. (2023a), Kunc et al. (2024)
20	*Rubus idaeus* subsp. *idaeus*	Fruit Leaf Shoot Trunk	Dry	cold, fever, flu, digestive disorders, uterine relaxant	Food industries -especially the berries-for juice, jam, wine and milk shake	Infusions Water or alcoholic extracts	Bowen-Forbes et al. (2010), Krauze-Baranowska et al. (2014), Hsin et al. (2017), Xu et al. (2017), Cyboran-Mikołajczyk et al. (2022)
21	*Ruscus aculeatus*	Fruit Leaf Root Stem	Dry -Fresh	Cardiovascular diseases, venous fragility, varicose veins, haemorrhoids, atherosclerosis, vasculitis, chronic venous insufficiency, hypotensive blood vessels, hypoxia, improving capillaries, edema, diabetes, skin diseases, warts, chilblains, eczema, kidney stones, nephritis, colitis, diarrhoea, arthritis, boils, cold, mastitis	-	Decoctions Extracts Powder	Longo and Vasapollo (2005), Hadžifejzović et al. (2013), Lichota et al. (2019), Michalak et al. (2023)
22	*Sambucus ebulus*	Flower Fruit Leaf Root	Dry - Fresh	Fever, infections, gout, cough, burns	Food industry as beverages, jam and syrups	Insect spray repellent (extract) Medicinal organs used as orally or locally for the treatment of disease as - Decoctions - Infusions - Poultice - Tea	Shokrzadeh and Saravi (2010), Popescu et al. (2014), Zahmanov et al. (2015), Kaya et al. (2019), Păvăloiu et al. (2019), Motevalli-Haghi et al. (2020)
23	*Sambucus racemosa*	Flower Fruit Leaf	Dry – Fresh	Immunostimulant, cardiovascular diseases, infectious, cough, influenza, bronchitis, fever, catarrah, astma, arthritis, viral infections	Food industry as juices, jam, jelly, syrups, liqueurs, and yoghurts	Infusions Powder	Masuda et al. (2012), Mikulic-Petkovsek et al. (2014), Mikulic-Petkovsek et al. (2015), Todorovic et al. (2017), Choi et al. (2024)
24	*Vaccinium myrtillus*	Flower Fruit Leaf	Dry -Fresh	Ocular health, vasculopathy, biliary disorders, bladder stones, coughs, scurvy, lung tuberculosis, diabetes, vascular permeability, night vision improving, hypertension, obesity, cardiovascular diseases, high blood pressure, gallstones, heart diseases, reducing chlolesterol, chronic illness, atherosclerosis, rheumatoid arthritis, digestive disorders, urinary tract disorders, eye inflammation, colitis, fever, cold, respiratory inflammation, sun protection factor, ocular dysfunction	Fruits eat as food Fruit Industry as juices, jam, liqueurs, pies, yoghurts, tarts and beverages	Decoctions Infusions	Ferlemi and Lamari (2016), Chehri et al. (2022), Karcheva-Bahchevanska et al. (2023), Martău et al. (2023), Cvetkova et al. (2024), Mercado et al. (2025)
25	*Vaccinium vitis-idaea*	Fruit Leaf Root Shoot	Dry - Fresh	Cystitis, diabetes, hypertension, GI disorders	Fruits eat as food Fruits are used in jams, juices, vines, pies	Infusions Powder	Pop et al. (2015), Shamilov et al. (2020), Vilkickyte et al. (2022), Pärnänen et al. (2024)
26	*Viscum album*	Flower Fruit Leaf Stem	Dry - Fresh	Spleen diseases, epilepsy, diabetic, wounds-healing, menstruation complaints, rheumatism, arthritis, hypertension, fetal aestlessness, liver and kidneys inflammation, malignant tumors, cardiovascular diseases, costipation, internal haemorrhages, stomach ulcers, skin diseases, thoracic duct injury after neck surgery	-	Extracts in oncology therapy (orally or subcutaneous and off-label intravenous injections - acupuncture)	Hajtó et al. (2011), Song et al. (2021a), Thronicke et al. (2022), Nicoletti (2023)
27	*Sambucus nigra*	Flower Fruit Leaf Root	Dry - Fresh	Fever Cold Infections, respiratory issues digestive comfort	Food industry as juices, syrups, jams, jellies, pies, desserts, yoghurt, ice creams, alcoholic beverages and natural colorants	Capsule Cordials Extracts Infusions Juices Powder Teas	Sidor and Gramza-Michałowska (2015), Petruţ et al. (2017), Porter and Bode (2017), Asgary and Pouramini (2022), Corrado et al. (2023), Stępień et al. (2023), Merecz-Sadowska et al. (2024)
Trees							
1	*Abies alba*	Bark Branch Cone; Knot Leaf Seed Shoot Woody parts	Dry - Fresh	Respiratory issues, skin wounds arthrosis cough, colds	-	Balsamic Decoctions Fragrances and bath products Infusions Inhalant products Lotion Ointments Syrup	Karkabounas et al. (2000), Ancuceanu et al. (2023)
2	*Aesculus hippocastanum*	Bark Flower Leaf Seed Stem	Dry	Fever, hemorrhoid cream, vascular skin cream, rheumatism, rectal complains, gastrointestinal disorders, post-operative edema, chronic venous insufficiency, varicose veins, skin protection	Potential preservation agent in food industry or packaging systems	Cream Oil Extract	Jarzębski et al. (2019), Čukanović et al. (2020), Owczarek et al. (2021), Babich et al. (2024)
3	*Betula pendula*	Bark Leaf	Dry	Rheumatism, gout, melanin synthesis disorders, diuretic, renal inflammation, blood and liver purification, fever, influenzal infections, stomach disorders, arthritis, kidney stones, rash, hepatitis, intestinal worms, scurvy, hair growth promoting, freckles	Food industry as beverages, vinegar, alcoholic drinks (beer), and sugar	Bath Birch sap Decoction Direct ingestion Foment Juice Tea infusion	Rastogi et al. (2015), Ostapiuk et al. (2021)
4	*Castanea sativa*	Bark Flower Fruit Leaf Seed	Dry	Diarrhea, gastrointestinal issues, hypertension	- Natural Food preservatives - Feed to increase the egg, broiler chickens, meat quality	-	Živković et al. (2009b), 2009a; Jukić et al. (2014), Brizi et al. (2016), Žitek Makoter et al. (2024)
5	*Fagus sylvatica*	Bark Leaf	Dry	Cough, bronchodilation, thrombosis, osteoarthritis	-	Decoction	Sivová et al. (2016), Coşarcă et al. (2019), Tanase et al. (2019)
6	*Fraxinus excelsior*	Bark Bud Leaf Seed	Dry	Rheumatism, neuralgia, gout	-	Extract	López-Carreras et al. (2013), Sarfraz et al. (2017), Iranpanah et al. (2024)
7	*Fraxinus ornus*	Bark Flower Leaf	Dry	Wound healing, inflammation, arthritis, dysentery, pediculosis, nutritive, articular pain, rheumatism	Food industry to product Manna	Decoction Exudates called Manna Infusion	Al-Snail (2018), Čopra-Janićijević et al. (2024)
8	*Juniperus communis*	Fruit	Dry	Anxiety, bronchitis, colic, coughts, stomachic, dyspesia, cystitis, arthritis, gout, inflammatory conditions, skin infection, itching, psoriasis, hyperpigmentation	- Food beverages as alcoholic drinks, liqueurs and gin -Food industry as spice for dish, and natural preservatives	Oil and ointments Extracts	Cioanca et al. (2014), Jegal et al. (2017), Tang et al. (2019), Čmiková et al. (2024)
9	*Larix decidua*	Bark Bud Leaf	Dry - Fresh	Wound healing, bronchitis, rheumatism	-	Balm Extract by hydrodistillation (Venice Turpentine) Ointments Perfumes for aromatherapy Powder Resin as cream/oil for internal and external use	Salem et al. (2016), Baldan et al. (2017), Batista et al. (2022), Goels et al. (2022)
10	*Picea abies*	Bark Bud Leaf	Dry - Fresh	Catarrhal diseases, antibiotics, cough, wound-healing, skin diseases, fever, skin regeneration, skin ulcers, human infections, irritation	Food industries as jams, syrups, and beverages	Baths Creams Oil Ointments Resin Resin boiled in fat Syrup Tea	Rautio et al. (2007), Radulescu et al. (2011), Kreps et al. (2017), Tanase et al. (2021), Goels et al. (2022), Sandulovici et al. (2024)
11	*Pinus cembra*	Bark Fruit Leaf Seed Shoot	Dry	Respiratory, circulation, diabetes	-The seed is used as pine nuts - Food alcoholic beverages (liqueurs)	Fragrances for aromatherapy; Oil Ointments	Apetrei et al. (2011), Lis et al. (2017), Nikolić et al. (2018), Chizzola and Müllner (2021), Alperth et al. (2023), Coman et al. (2023)
12	*Pinus mugo*	Bark Bud Fruit Leaf Shoot	Dry - Fresh	Cough, throat inflammation, diuresis, skin problems, atherosclerosis, stroke, wound-healing, cold	-Natural food preservatives - Food alcoholic beverages (liqueurs) - Food industries as a spice, pine nuts	Bath for soaps, detergents, and household cleaning supplies Deodorants Fragrances for aromatherapy Inhaling products Infusion Oil Syrup	Grassmann et al. (2003), Stevanovic et al. (2005), Venditti et al. (2013), Hajdari et al. (2015), Lis et al. (2019), Popescu et al. (2022)
13	*Pinus sylvestris*	Bark Bud Fruit Leaf Shoot	Dry - Fresh	Wound-healing, asthma, diarrhea, liver disease, respiratory infections, cough	-	Oil Ointments	Karonen et al. (2004), Laavola et al. (2015), Duțu et al. (2022); Intas (2023), Macovei et al. (2023)
14	*Populus tremula*	Bark Bud Leaf Shoot Trunk	Dry	Respiratory issues, fever, skin issues	Propolis from resin harvested by beees	Extracts	Gundermann et al. (2020); Meyer et al. (1995), Neacsu et al. (2007), Keefover-Ring et al. (2014), Kis et al. (2021), Tsvetkov et al. (2022)
15	*Prunus avium*	Flower Fruit Leaf Stem (Peduncle)	Dry – Fresh	Urinary tract infections, urinary toxin elimination, diabetes, gouty arthritis, sleep regulation, obesity, hyperthension, cardiovascular disease, blood, gastrointestinal malignancies, atherosclerosis	Food industry as candies, jellies, juices, jam, liqueurs, and fresh-cut fruits	Decoction Infusion	Beconcini et al. (2020), Faienza et al. (2020), Nunes et al. (2021), Barreto-Peixoto et al. (2023)
16	*Prunus spinosa*	Flower Fruit Leaf	Dry - Fresh	Diabetes, spasm, blood purifying, cardiovascular diseases, hypertension, oral and pharyngeal mucosa inflammatory, mouth-wash, edema, kidney disease, stomach pain, wound-healing	- Food industry as dietary supplements, novel green preservatives and jams - Food beverages as liqueurs, fruit juice	Decoction Extract Mouth-wash	Aliyazicioglu et al. (2015), Băbălău-Fuss et al. (2021), Coppari et al. (2021), Temiz and Okumus (2022), Negrean et al. (2023)
17	*Pyrus pyraster*	Bud Flower Fruit Leaf	Dry - Fresh	Urinary therapy, diabetes, skin disorders	Food industry as juice, jam, fresh fruit, snacks	Potentially novel products as cosmetics and drugs	Wang et al. (2023), Elankathirselvan et al. (2024), Molinu et al. (2024), Samkaria et al. (2024)
18	*Quercus petraea*	Bark Fruit Seed	Dry	Wound-healing, throat diseases, bronchitis, diabetes, tonsillitis, gonorrhea, chronic diarrhea, dysentery, intermittent fevers, haemorrhages, skin eruptions, sweat feet, piles, genital inflammation, mouth infections, gallbladder disorders, spleen disorders	Source of food for animal husbandery	Decoction Ointment	Tumen et al. (2018), Coman et al. (2023), Nisca et al. (2023)
19	*Quercus pubescens*	Bark	Dry	Wound-healing, throat diseases, stomach disorders, constipation, flatulance, bronchitis, diabetes, tonsillitis, skin eruptions, sweat feet, piles, genital inflammation, mouth infections, gall bladder disorders	Source of food for animal husbandery	-	Nisca et al. (2023)
20	*Quercus robur*	Bark Bud	Dry	Gastrointestinal disorders, gynecological diseases, kidney disorders, wound-healing, infectious diseases	Source of food for animal husbandery	-	Ryabov et al. (2021), Teterovska et al. (2023)
21	*Salix alba*	Bark Leaf	Dry	Fever, pain, rheumatic disorders	Food beverages with higher functional value	Decoctions Infusions Tea	Harbourne et al. (2009), Kenstavičienė et al. (2009), Qadir et al. (2020), Aleman et al. (2023), Gandhi et al. (2023), Sandulovici et al. (2024)
22	*Salix caprea*	Bark Bud Flower Leaf	Dry	Heartburn, stomach problems, pain, inflammatory diseases, obesity, fever, reproductive disorders, rheumatoid arthritis, hemorrhages, gout, intestinal diseases	-	Extracts with water and/or EtOH Tea	Pohjamo et al. (2003), Alam et al. (2006), Moohammadnor et al. (2010), Calorio et al. (2017), Gligorić et al. (2019b), Jang et al. (2022)
23	*Salix pentandra*	Bark	Dry	Rheumatic disturbances, infections, headaches, fever, arthritis, pain, gonarthrosis, coxarthrosis, spasms	-	Extracts	Förster et al. (2021)
24	*Salix purpurea*	Bark Leaf	Dry	Pain, fever, inflammation, cardiovascular diseases, obesity	-	Extracts	Pobłocka-Olech et al. (2010), Sulima et al. (2017), Gligorić et al. (2019a), Köhler et al. (2020)
25	*Sorbus aucuparia*	Flower Fruit	Dry - Fresh	GI disorders, cardiovascular diseases, fever, atherosclerosis	Food industry as fruit pomace, juices, jams, jellies, alcoholicbeverage, and confectionary	Extracts	Šavikin et al. (2017), Stanković et al. (2019), Rutkowska et al. (2021), 2023; Aurori et al. (2023), 2024
26	*Tilia cordata*	Flower Fruit Leaf	Dry	Colds, cough, flu, fever, bronchitis, catarrh, infectious diseases, gastrointestinal disorders, hypertension, spasms, diarrhea, gastrointestinal muscle inihibitory, anxia	Food industry as oil, foodstuffs, natural preservatives	Decoctions Extracts Infusions Oil Tea Tinctures	Al-Essa et al. (2007), Wilczak et al. (2013), Oniszczuk and Podgórski (2015), Cittan et al. (2018), Siger et al. (2021), Symma et al. (2021)
27	*Tilia platyphyllos*	Flower Fruit Leaf	Dry	Wound dressings, wounds-healing, colds, cough, runny noses, indigestion, high blood pressure, anxiety, hysteria, nervous vomiting, skin inflammation, skin redness, skin dry and sensitive, mental stress, migraine, liver and gall bladder disorders	Food industry as oil, foodstuffs, natural preservatives	Cream Decoctions Extracts Infusions Ointments Oil Tea Tinctures	Bardakci et al. (2020), Siger et al. (2021), Symma et al. (2021), Kędzierska et al. (2023)

Roots and rhizomes, although less frequently cited, also contribute to Alpine ethnopharmacology, particularly among species such as *Rubus idaeus* L. (Xu et al., 2017; Alinia-Ahandani et al., 2023). These parts are usually processed into decoctions or macerates to treat urogenital, hepatic, and dermatological disorders, reflecting their content of alkaloids, and saponins. Other organs, such as cones, seeds, and wood tissue, have specialized uses. For example, cones of *P. abies* (L.) H. Karst. and *P. sylvestris* L. are incorporated into expectorant syrups, while seeds from *J. communis* and *Sorbus domestica* may be used for digestive and metabolic support (Barros et al., 2011; Jarzycka et al., 2013; Alirezalu et al., 2018; Moldovan et al., 2021). The inner wood of certain species (*Rosa* x *damascena*) is sometimes distilled for essential oil production, known for antimicrobial and respiratory-stimulant effects (Nazıroğlu et al., 2013).

The selection and preparation of specific plant parts are closely linked to their traditional therapeutic targets. Application data, when mapped against the ICPC-2, shows that the majority of treatments address respiratory (R), digestive (D), musculoskeletal (L), and urinary (U) system disorders (World Health Organization, 2009). Additional indications include skin conditions (S), cardiovascular support (K), endocrine and metabolic regulation (T), and general or systemic tonics (A). The wide array of organ-specific applications underscores the pharmacological versatility of Alpine woody plants and reflects deep empirical knowledge of their phytochemical specificity (Petelka et al., 2020). This functional diversity also implies that conservation efforts should consider not only species identity but also intra-organ variability and phenological timing, to ensure both sustainable use and preservation of bioactive efficacy.

There is a comprehensive mapping of the medicinal uses of woody species along with the specific plant organs utilized, their state (dry or fresh), and associated therapeutic preparations (Table 4). Only traditional European medicinal uses are included in this table. Uses originating from non-European systems (e.g., Asian or Middle Eastern traditions) or derived solely from laboratory experiments were excluded. This ethnopharmacological compilation underscores the depth of empirical botanical knowledge embedded in traditional healthcare systems. For example, *Berberis vulgaris* has been extensively used for managing liver disease, coronary artery disorders, and metabolic syndromes, with modern research validating its hypoglycemic and hypolipidemic effects (Imenshahidi and Hosseinzadeh, 2016; Rahimi-Madiseh et al., 2017). Similarly, *V. myrtillus* has demonstrated traditional applications in treating vascular and ocular disorders, and its high anthocyanin content has been linked to vascular protection and anti-inflammatory properties (Ferlemi and Lamari, 2016; Martău et al., 2023). *Rosa canina*, widely employed for gastrointestinal and skin disorders, has been shown to exert antioxidant and anti-inflammatory activities attributed to its high polyphenolic content (Ayati et al., 2021; Wang S. et al., 2022). These examples reflect a strong alignment between traditional uses and pharmacological findings, underscoring the relevance of such species for further phytochemical and pharmacological investigations.

### 3.3 Therapeutic applications and clinical targeting

Building directly on the ethnobotanical uses summarized in Sections 3.1–3.2, we next examined pharmacological data only for the same 54 Alpine woody species, in order to evaluate how far preclinical and clinical evidence corroborates traditional indications. Among the most frequently targeted systems, the respiratory tract, gastrointestinal system, dermatological conditions, metabolic disorders, and immune-related dysfunctions figure prominently (Duțu et al., 2022; Barreto-Peixoto et al., 2023). These therapeutic domains are commonly associated with chronic or recurrent conditions in high-altitude populations, and the ethnopharmacological response embedded in Alpine herbal knowledge reflects both environmental exposure and long-standing adaptive strategies (Petelka et al., 2020).

The respiratory system is a primary target for many Alpine woody taxa, particularly coniferous species such as *Abies alba* and *P. mugo* (Table 4). These effects are relevant for treating bronchitis, sinusitis, chronic obstructive pulmonary disease (COPD), and upper respiratory tract infections. Extracts from *Hedera helix* L., and *Sambucus nigra* L. further extend the therapeutic range through antitussive and immunostimulatory effects, which are supported by both *in vitro* viral inhibition studies and clinical applications in phytotherapeutic formulations (Akhlaghi et al., 2022; Corrado et al., 2023; Qabaha et al., 2023).

Musculoskeletal and inflammatory disorders, including rheumatism, gout, and arthritis, are commonly addressed through bark and bud preparations from species such as *S. caprea* (Moohammadnor et al., 2010). These taxa contain flavonoids, and tannins that exert anti-inflammatory and analgesic effects via inhibition of cyclooxygenase pathways and modulation of inflammatory cytokines (Cucu et al., 2022; Alhujaily, 2023). In traditional applications, decoctions, tinctures, and topical liniments derived from these species are employed to manage joint stiffness, muscular pain, and local inflammation. *Genista tinctoria* and *Ononis spinosa* while less frequently cited, contribute unique alkaloids and isoflavones with demonstrated anti-nociceptive and anti-edematous activity in pharmacological models (Rigano et al., 2009; Rigano et al., 2010; Abbas and Jaffal, 2019).

In the dermatological domain, Alpine woody species provide a wide array of therapeutic agents for treating wounds, burns, skin infections, and inflammatory dermatoses. Extracts of *Calluna vulgaris, Ribes petraeum* and *R. canina* have been tested for wound-healing efficacy, antioxidant action, and antimicrobial activity (Šikšnianas et al., 2013; Kendir et al., 2019; Sun et al., 2021). Their bioactivity is attributed to polyphenols, proanthocyanidins, and organic acids that modulate oxidative stress and promote dermal regeneration. Notably, hydrogels derived from *Aesculus hippocastanum* have demonstrated efficacy in reducing erythema, promoting fibroblast proliferation, and accelerating re-epithelialization in experimental models, supporting their integration into cosmeceutical and clinical dermatology formulations (Michalak et al., 2023).

To systematically contextualize the therapeutic spectrum of these Alpine woody species, their ethnopharmacological indications were mapped using the World Health Organization’s International Classification of Primary Care (ICPC-2, second edition). This clinical framework enables an evidence-aligned categorization of plant-based uses into modern therapeutic domains.

A summary is shown from the ICPC-2 category assignments for each species, highlighting the predominance of dermatological (S), digestive (D), musculoskeletal (L), cardiovascular (K), and metabolic/endocrine (T) targets (Table 5). The high frequency of species affecting multiple systems underscores the multifunctionality of Alpine ethnobotanical knowledge. Therapeutic strategies for metabolic disorders, particularly type 2 diabetes and dyslipidemia, are also well-represented among the woody Alpine flora. Extracts from *B. vulgaris, C. avellana*, *Crataegus monogyna*, and *V. myrtillus* exhibit hypoglycemic, hypolipidemic, and insulin-sensitizing effects (Imenshahidi and Hosseinzadeh, 2016). The active constituents include isoquinoline alkaloids such as berberine, flavonoids, and anthocyanins, many of which act through inhibition of α-glucosidase, modulation of AMP-activated protein kinase (AMPK) pathways, and protection of pancreatic β-cell function. Controlled *in vivo* studies and preliminary human trials have confirmed these bioactivities, positioning these taxa as promising candidates for adjunctive management of diabetes and associated cardiometabolic syndromes (Pop et al., 2015).

**TABLE 5:** Therapeutic classifications were derived from an evidence-based synthesis of published ethnopharmacological uses and assigned to ICPC-2 domains according to WHO guidelines (World Health Organization, 2009).

No.	Scientific name	ICPC-2 categories^a^																
		A	B	D	F	H	K	L	N	P	R	S	T	U	X	Y	Z	W
Shrubs																		
1	*Arctostaphylos uva-ursi*	​	​	​	​	​	​	​	​	​	​	​	​	X	​	​	​	​
2	*Berberis vulgaris*	X	X	X	X	X	X	X	X	X	X	X	X	X	​	X	​	X
3	*Calluna vulgaris*	​	X	X	​	​	​	​	X	X	​	X	​	X	​	​	​	X
4	*Corylus avellana*	X	X	​	​	​	X	​	X	​	​	​	​	​	​	​	​	X
5	*Crataegus monogyna*	​	X	X	​	​	X	X	X	X	X	X	X	​	​	X	​	X
6	*Erica carnea* subsp. *carnea*	​	X	​	​	​	​	​	​	​	​	X	​	X	​	​	X	​
7	*Genista tinctoria*	X	X	​	​	​	X	X	X	X	​	​	X	X	​	X	X	X
8	*Hedera helix*	X	​	​	​	​	​	​	​	​	X	​	X	​	​	X	​	​
9	*Hippophae rhamnoides* subsp. *fluviatilis*	X	X	X	​	​	X	X	X	​	X	X	​	​	​	​	​	​
10	*Humulus lupulus*	​	​	X	​	​	X	X	X	X	​	​	X	​	​	X	X	X
11	*Myricaria germanica*	​	X	X	​	​	​	X	​	​	X	​	​	​	​	X	X	​
12	*Ononis spinosa*	X	X	X	​	​	​	X	​	​	​	X	X	X	​	X	​	​
13	*Ribes petraeum*	X	X	X	X	​	X	X	​	​	X	X	X	X	​	X	​	​
14	*Rosa canina*	X	X	X	​	X	X	X	X	X	X	X	X	X	​	X	​	X
15	*Rosa corymbifera*	X	X	X	​	​	X	X	X	​	​	X	X	X	​	​	​	​
16	*Rosa x damascena*	X	X	X	​	​	X	X	X	X	X	X	X	​	X	X	​	X
17	*Rosa montana*	X	​	​	​	​	X	​	​	​	​	​	X	​	​	​	​	​
18	*Rosa pendulina*	X	X	X	​	​	X	X	X	X	​	X	X	X	​	​	​	X
19	*Rubus idaeus* subsp. *idaeus*	X	X	X	​	​	​	X	​	​	X	X	​	X	​	X	​	​
20	*Ruscus aculeatus*	X	X	X	​	​	X	X	​	​	X	X	X	X	​	​	​	​
21	*Sambucus ebulus*	X	X	X	​	​	​	X	X	​	X	X	​	X	​	​	​	​
22	*Sambucus racemosa*	X	X	​	​	​	X	X	​	​	X	​	​	​	​	​	​	​
23	*Vaccinium myrtillus*	X	X	X	X	​	X	X	X	X	X	X	X	X	​	X	​	X
24	*Vaccinium vitis-idaea*	X	X	X	X	​	X	X	X	X	​	X	X	X	​	​	​	​
25	*Viscum album*	X	X	X	​	​	X	X	X	​	X	X	X	X	X	X	​	​
26	*Sambucus nigra*	X	X	X	X	​	X	​	X	X	X	X	​	​	​	​	​	X
27	*Juniperus communis*	X	X	X	​	​	​	X	X	X	X	X	​	X	​	​	​	X
Total shrubs		21	23	20	5	2	18	20	17	12	16	19	16	17	2	13	4	12
Trees																		
1	*Abies alba*	X	​	X	​	​	​	​	​	​	​	X	X	​	​	​	​	​
2	*Aesculus hippocastanum*	X	X	X	​	​	​	X	​	​	​	X	​	​	​	​	​	​
3	*Betula pendula*	X	X	X	​	​	​	X	​	​	X	X	​	X	​	​	​	​
4	*Castanea sativa*	​	X	X	​	​	X	​	X	X	X	X	X	​	​	​	​	X
5	*Fagus sylvatica*	X	X	X	​	​	X	X	​	​	X	X	X	​	​	​	​	​
6	*Fraxinus excelsior*	X	X	X	​	​	​	X	X	​	X	X	​	X	​	X	​	​
7	*Fraxinus ornus*	X	​	X	​	​	​	X	​	​	​	X	X	​	​	​	​	​
8	*Larix decidua*	X	X	X	X	​	​	X	X	X	X	X	​	X	​	​	​	X
9	*Picea abies*	X	.	​	​	​	​	​	​	X	X	X	​	​	​	​	​	X
10	*Pinus cembra*	​	X	​	​	​	X	X	X	X	X	X	​	​	​	X	​	X
11	*Pinus mugo*	​	​	​	​	​	X	X	X	​	X	X	​	X	​	​	​	​
12	*Pinus sylvestris*	X	​	X	​	​	​	X	​	​	X	X	​	​	​	X	​	​
13	*Populus tremula*	X	X	X	​	​	​	X	​	​	X	X	X	​	​	​	​	​
14	*Prunus avium*	​	X	X	​	​	X	X	X	X	​	​	X	X	​	​	​	X
15	*Prunus spinosa*	X	X	X	​	​	X	X	​	​	X	X	X	X	​	​	​	​
16	*Pyrus pyraster*	X	X	X	​	​	​	​	​	​	X	X	X	X	​	X	​	​
17	*Quercus petraea*	X	X	X	​	​	​	​	​	​	X	X	X	​	​	X	X	​
18	*Quercus pubescens*	X	X	X	​	​	​	​	​	​	X	X	X	​	​	X	X	​
19	*Quercus robur*	X	X	X	​	​	​	​	​	​	​	X	​	X	​	X	X	​
20	*Salix alba*	X	X	​	​	​	X	X	X	X	X	X	​	​	​	​	​	X
21	*Salix caprea*	X	X	X	​	​	X	X	X	X	​	X	​	​	X	​	​	X
22	*Salix pentandra*	X	​	​	​	​	​	X	X	​	​	​	​	​	​	​	​	​
23	*Salix purpurea*	X	​	​	​	​	X	X	X	X	​	​	​	​	​	​	​	X
24	*Sorbus aucuparia*	X	X	X	​	​	X	​	​	​	X	X	X	X	​	​	​	​
25	*Tilia cordata*	X	X	X	​	​	​	X	X	X	X	​	​	​	​	​	​	X
26	*Tilia platyphyllos*	​	X	X	​	​	​	​	X	X	X	X	​	​	​	​	​	X
27	*Ilex aquifolium*	X	X	X	​	​	​	​	X	​	​	​	​	X	​	X	​	​
Total trees		22	20	21	2	0	10	17	13	10	18	22	11	10	1	8	4	10
Total shrubs and trees		43	43	41	7	2	28	37	30	22	34	41	27	27	3	21	8	22

^a^ The detailed meaning of ICPC-2, chapters was reported in Table 1 of Material and methods section.

In the context of cardiovascular protection, species such as *C. monogyna, Ruscus aculeatus*, and *S. aucuparia* have gained recognition for their vasoprotective, hypotensive, and anti-atherogenic effects (Barros et al., 2011; Hadžifejzović et al., 2013; Alirezalu et al., 2018; Moldovan et al., 2021). The presence of procyanidins, quercetin derivatives, and phytosterols is associated with endothelial modulation, improved lipid profiles, and inhibition of platelet aggregation. Clinical and pharmacodynamic studies have demonstrated reductions in blood pressure, low-density protein (LDL) levels, and markers of vascular inflammation, especially with long-term administration of polyphenol-rich extracts.

From the perspective of traditional European medicine, Alpine woody species were not historically employed as specific remedies for malignant diseases. However, several taxa that are firmly embedded in European ethnomedicine have been investigated in modern pharmacological studies for their potential anticancer effects. In particular, extracts and isolated compounds from *F. excelsior* L., *Q. robur* L. and *Viscum album* L. have shown cytotoxic, pro-apoptotic and anti-proliferative activities in preclinical models of breast, prostate and colorectal cancers (Berkoz et al., 2019; Ryabov et al., 2021; Sarfraz et al., 2017; Thronicke et al., 2022). These effects are largely driven by oxidative-stress modulation, inhibition of angiogenesis and interference with tumour cell-cycle progression. They should therefore be interpreted as contemporary biomedical extensions of a traditional European materia medica, rather than as historical ethnomedical indications for cancer treatment. Although clinical validation remains limited, the mechanistic basis for these activities aligns with current anticancer pharmacology and identifies these species as candidates for future drug-discovery pipelines (Berkoz et al., 2019). Compounds isolated from *F. excelsior* L. (Sarfraz et al., 2017)*, Q. robur* L. (Ryabov et al., 2021), and *V. album* L. (Thronicke et al., 2022) have shown activity in preclinical models of breast, prostate, and colorectal cancers. These effects are largely driven by oxidative stress modulation, inhibition of angiogenesis, and interference with tumor cell cycle progression. Although clinical validation remains limited, the mechanistic basis for these activities aligns with contemporary anticancer pharmacology, indicating significant potential for future exploration (Petrovska, 2012; Alrhmoun et al., 2025).

Emerging evidence also supports the neuroprotective and cognitive-enhancing potential of certain Alpine woody plants. *Corylus avellana* and *Humulus lupulus* contain amino acids and bitter acids that affect neurotransmission, neuroinflammation, and synaptic plasticity, potentially improving memory and stress resilience (Nuutinen, 2018; Cabo et al., 2021). Similarly, *Rosa damascena* Mill. essential oils have been investigated for anxiolytic and sedative properties, with preliminary clinical trials reporting improvements in sleep quality and anxiety indices (Nazıroğlu et al., 2013).

Several species also contribute to urogenital and hormonal regulation, particularly *Arctostaphylos uva-ursi* (L.) Spreng., *G. tinctoria* L., and *R. idaeus* L. (Mohd Azman et al., 2016; Cyboran-Mikołajczyk et al., 2022). Arbutin-rich leaves of *A. uva-ursi* exhibit urinary antiseptic activity, while genistein in *G. tinctoria* acts as a phytoestrogen, modulating hormonal balance in menopausal and reproductive disorders. These uses are consistent with their traditional indications (Table 5) and have been partially validated by pharmacological assays and hormone receptor binding studies.

A comprehensive overview of the pharmacological activities of these woody Alpine species is provided (Table 6). This summary highlights the solvent systems used for bioactive extraction, the relevant plant parts, phytochemical classes, and pharmacodynamic mechanisms evidenced in both *in-vitro* and *in-vivo* studies. For instance, *B. vulgaris* extracts-rich in alkaloids and phenolics-exhibited calcium channel-blocking and cholesterol-regulating effects through berbamine and berberine, respectively (Imenshahidi and Hosseinzadeh, 2016; Behravan et al., 2019). Similarly, *C. vulgaris* demonstrated hepatoprotective effects by upregulating antioxidant enzymes and reducing hepatic inflammation markers such as COX-2 and TNF-α (Cucu et al., 2022). *Vaccinium myrtillus* showed multifunctional bioactivities, including α-glucosidase inhibition, antioxidant cardioprotection, and suppression of pro-inflammatory cytokines (Ferlemi and Lamari, 2016). These examples underscore the mechanistic diversity and therapeutic potential of alpine woody species, validating their traditional applications and highlighting their relevance in modern phytomedicine.

**TABLE 6:** Summary of pharmacological activities of plant extracts from different parts of woody medicinal species.

Species	Extract solvent	Plant parts	Class	*In vitro-* *in vivo*	Mechanism of action	References
*Arctostaphylos uva-ursi*	EtOH (70%)	Leaf	Phenols Flavonoids; Tannins	*In vivo*	Arbutin is absorbed in the intestine and hydrolyzed by gut microbes or the liver into hydroquinone conjugates, which are excreted in urine	de Arriba et al. (2013), Olennikov and Chekhirova (2013), Kurkin et al. (2018), Song et al. (2021b)
*Berberis vulgaris*	Water EtOH MeOH Butanol Ethyl acetate; n-hexane	Bark Fruit Leaf Root Shoot	Alkaloids Anthocyanins Minerals Organic Acids Phenols Sugars and poliysaccharides Tannins Terpenoids Vitamins	*In vivo*	Berbamine blocks calcium channels and inhibits lipid peroxidation in red blood cells. Berberine upregulates hepatic receptors involved in cholesterol binding and excretion. Vitamin C enhances serotonin synthesis, boosts liver ferritin, and prevents carcinogenic nitrosamine formation	Imenshahidi and Hosseinzadeh (2016), 2019; Rahimi-Madiseh et al. (2017), Behravan et al. (2019), Rahman et al. (2022), Ivan et al. (2024)
*Calluna vulgaris*	EtOH	Flower Leaf Shoot	Flavonoids Phenols Terpenoids	*In vivo*	The extracts showed hepatoprotective effects by enhancing hepatic antioxidant enzymes and reducing inflammatory markers such as COX-2, iNOS, and TNF-α. Overall, the phytocompounds exhibited strong antioxidant activity against ROS.	Perde-Schrepler et al. (2011), Bekkai et al. (2022), Cucu et al. (2022), Alhujaily (2023)
*Corylus avellana*	Acetone MeOH EtOH Ethyl acetate; n-hexane Water	Bark Bud Fruit Leaf Male Flower Seed	Amino Acids Flavonoids Fatty Acids Phenols Phytosterols Terpenoids Vitamins	*In vivo*	Their antioxidant activity reduces ROS-related inflammation, modulates inflammatory gene expression, inhibits iron-mediated reactions, supports macrophage activity, and lowers cholesterol synthesis and blood glucose levels	Najda and Gantner (2012), Cappelli et al. (2018), Cabo et al. (2021), Zhao et al. (2023)
*Crataegus monogyna*	EtOH Chloroform Water	Leaf Flower Fruit	Carotenoids Fatty Acids Flavonoids Phenols Tocopherols; Vitamins	*In vitro* *In vivo*	Exhibits α-glucosidase inhibition to reduce glucose absorption and broad antioxidant activity against disease-related reactive species	Barros et al. (2011), Jarzycka et al. (2013), Alirezalu et al. (2018), Moldovan et al. (2021)
*Erica carnea* subsp. *carnea*	EtOH	Leaf Flower	Flavonoids; Phenols	*In vitro* *In vivo*	High antioxidant capacity inhibits bacterial and tumor cell proliferation. In dermatology, it reduces inflammation through ROS scavenging, iron chelation, and glutathione-S-transferase induction	Pavlović et al. (2013), Veličković et al. (2017)
*Genista tinctoria*	Chloroform MeOH Petroleum ether	Flower Leaf Root Seed Stem	Carbonylic acids Fatty Acids Flavonoids	*In vitro*	Induces tumor cell necrosis by disrupting the cytoplasmic membrane and releasing cytotoxic factors. Flavonoid-like compounds also exhibit anti-estrogenic effects by blocking estrogen receptors	Rigano et al. (2009), 2010; Sharifi-Rad et al. (2021)
*Hedera helix*	EtOH	Fruit Leaf Stem	Flavonoids Phenols Saponins Terpenoids	*In vitro* *In vivo*	Saponins disrupt membrane integrity, exerting antiproliferative effects across all *Leishmania* growth phases. Hederasaponin B inhibits influenza VP2 protein synthesis, blocking viral capsid formation. Hederin enhances β2-adrenergic responsiveness in lung cells by increasing receptor binding and cAMP levels, promoting surfactant secretion	Delmas et al. (2000), Hong et al. (2015), Akhlaghi et al. (2022), Gavrila et al. (2022), Osama et al. (2023), Qabaha et al. (2023)
*Hippophae rhamnoides* subsp. *fluviatilis*	Butanol EtOH Hexane Water	Branch Fruit Leaf Root Seed	Carotenoids Fatty Acids Flavonoids Organic Acids Phenols Phytosterols Proteins Tannins Vitamins	*In vitro* *In vivo*	The extract exhibits hepatoprotective effects by reducing lipid peroxidation and liver enzymes linked to fatty liver injury. It scavenges ROS associated with cardiac damage and modulates calcium and VEGF signaling during stroke. Additionally, it promotes wound healing by enhancing collagen synthesis, cell regeneration, angiogenesis, and re-epithelialization	Buyukokuroglu and Gulcin (2009), Suryakumar and Gupta (2011), Kong et al. (2020), Żuchowski (2023), Ma et al. (2024)
*Humulus lupulus*	EtOH	Flower Fruit Leaf Shoot	Flavonoids Organic Acids Terpenoids	*In vitro* *In vivo*	The extract promotes apoptosis and inhibits cancer cell migration. It also enhances hippocampal dopamine levels and activates D1 receptors, supporting neurocognitive function	Nuutinen (2018), Lin et al. (2019), Yan et al. (2021), Bektur et al. (2022)
*Ilex aquifolium*	2-undecanone Water	Leaf	Flavonoids Fatty Acids Organic Acids Phenols Phytosterols Saponins Terpenoids	*In vitro* *In vivo*	Antiviral effects involve blocking viral proteins to prevent cell attachment. At the genetic level, the extract downregulates LOX-1 (linked to atherosclerosis) and upregulates ACAT-1, promoting beneficial cholesterol regulation	Paluch et al. (2021), Pachura et al. (2022), 2024
*Myricaria germanica*	EtOH MeOH Water	Bark Leaf	Flavonoids Organic Acids Phenols	*In vitro* *In vivo*	The extract enhances immune activity and may serve as a vaccine co-adjuvant. It reduces collagen degradation and positively regulates RNA polymerase II in arthritis. Additionally, it exhibits cytotoxic effects against breast, liver, and prostate cancer cells	Nawwar et al. (2013), Liu et al. (2023), Wang et al. (2024)
*Ononis spinosa*	Acetone EtOH MeOH Water	Flower Leaf Root	Flavonoids Terpenoids	*In vivo*	The antinociceptive effect is linked to phospholipase A2 and TRPM3 inhibition, independent of ATP-sensitive K^+^ channels, opioids, or muscarinic receptors. Diuretic and urinary benefits arise from inhibition of human hyaluronidase-1 (Hyal-1)	Yõlmaz et al. (2006), Benedec et al. (2012), Addotey et al. (2018), Abbas and Jaffal (2019)
*Ribes petraeum*	MeOH	Fruit Leaf	Anthocyanins Fatty Acids Flavonoids Phenols Sugar and Polysaccharides Terpenoids	*In vivo* *In vitro*	In dermatology, healing effects involve enhanced re-epithelialization, fibroblast proliferation, collagen formation, immune cell recruitment, and angiogenesis. In ophthalmology, the extract reduces vitreous chamber and ocular enlargement, improves ocular blood flow, and slows glaucoma progression	Šikšnianas et al. (2013), Kendir et al. (2019), Sun et al. (2021)
*Rosa canina*	Acetone EtOH Ethyl acetate MeOH; n-hexane Water	Flower Fruit Leaf Root Seed Stem	Anthocyanins Carotenoids Fatty Acids Flavonoids Phenols Phytosterols Terpenoids Tannins Tocopherols Vitamins	*In vivo* *In vitro*	Anti-inflammatory effects stem from NF-κB pathway inhibition, while anticancer activity involves suppression of MAPK/AKT signaling and G0/G1 cell cycle arrest. The extract enhances collagen synthesis for skin repair, exhibits antioxidant protection against pulmonary diseases and rheumatism, and inhibits α-glucosidase, improving insulin sensitivity and pancreatic function for antidiabetic effects	Ayati et al. (2021), Băbălău-Fuss et al. (2021), Wang et al. (2022a), Demirci Kayiran et al. (2023), Negrean et al. (2023), Manzione et al. (2024), Saini et al. (2024)
*Rosa corymbifera*	MeOH Water	Fruit Seed	Anthocyanins Flavonoids Fatty Acids Phenols Tocopherols Vitamins	*In vitro* *In vivo*	Antioxidant activity arises from metal chelation, lipoxygenase inhibition, and free radical scavenging. The extract also shows antidiabetic effects by inhibiting carbohydrate-hydrolyzing enzymes and neuroprotective effects through cholinesterase inhibition	Kayahan et al. (2023), Kunc et al. (2023a)
*Rosa x damascena*	Acetone Chloroformic EtOH Ethyl Acetate MeOH Water	Flower	Flavonoids Organic Acids Phenols Terpenoids Vitamins	*In* *vivo* *In vitro*	The extract exhibits antidepressant and anxiolytic effects by inhibiting monoamine and serotonin reuptake. It improves memory by reducing MDA, increasing thiols, and inhibiting acetylcholinesterase. Anticonvulsant and sleep-enhancing effects involve GABA_A_ and benzodiazepine receptor modulation, reducing neuronal apoptosis. High antioxidant capacity contributes to antimicrobial and analgesic properties	Boskabady et al. (2011), Nayebi et al. (2017), Osama and Ikram (2018), Ramdan et al. (2021), Raeeszadeh et al. (2022), Yaghoobi et al. (2022)
*Rosa montana*	n-hexane	Fruit Seed	Fatty Acids Minerals Vitamins	*-*	High levels of fatty acids and vitamins contribute to strong antioxidant activity against reactive species and exert cytotoxic effects	Yilmaz et al. (2011), Hatipoglu and Ak (2023)
*Rosa pendulina*	Acetonitrile Formic acid MeOH Meta-phosphoric acid Water	Flower Fruit Seed	Anthocyanins Flavonoids Organic Acids Phenols Terpenoids Tannins Vitamins	*-*	Abundant phytocompounds-tannins, phenols, and flavonoids-provide potent antioxidant activity beneficial in preventing and managing human diseases	Karczmarz et al. (2019), Kunc et al. (2022), Kunc et al. (2023b), Kunc et al. (2024)
*Rubus idaeus* subsp. *idaeus*	EtOH Ethyl Acetate MeOH Water with SO_2_ (200 ppm)	Fruit Leaf Shoot Trunk	Anthocyanins Flavonoids Lignans Phenols Tannins	*In vitro*	The extract preserves erythrocyte integrity by maintaining osmotic balance and membrane stability, while its antioxidant activity protects against lipid peroxidation and ROS-induced damage. Additionally, it inhibits nasopharyngeal carcinoma cell migration by downregulating MMP-2 via the ERK1/2 pathway, supporting chemotherapeutic and chemopreventive potential	Bowen-Forbes et al. (2010), Krauze-Baranowska et al. (2014), Hsin et al. (2017), Xu et al. (2017), Cyboran-Mikołajczyk et al. (2022)
*Ruscus aculeatus*	Glycerol Water	Fruit Leaf; Root Stem	Anthocyanins Flavonoids Phenols Saponins Vitamins	*In vitro*	Phytocompounds offer antioxidant protection by neutralizing free radicals. In dermatology, Ruscus extract regulates skin pH, promotes regeneration, and inhibits microbial growth. It supports vascular health by inhibiting elastase, enhancing circulation, and reducing hypertension and venous inflammation. In varicose veins, it improves venous outflow, limits leukocyte activation, and downregulates inflammatory mediators such as MMP-2, MMP-9, ECM enzymes, and TGF-β, preventing fibrosis	Longo and Vasapollo (2005), Hadžifejzović et al. (2013), Lichota et al. (2019), Michalak et al. (2023)
*Sambucus ebulus*	Ethyl acetate EtOH MeOH; n-hexane Water	Flower Fruit Leaf Root	Anthocyanins Alkanes Flavonoids Organic Acids Phenols Saponins Tannins Terpenoids	*In vivo* *In vitro*	Phytocompounds act as insect repellents by damaging insect gut epithelium. Their antioxidant properties neutralize free radicals and support anti-inflammatory, antimicrobial, and antiviral defenses. Anti-inflammatory effects may involve glucocorticoid signaling or interaction with serotonergic and tachykinin pathways. Anticancer activity includes cytotoxicity and inhibition of tumor vascularization	Shokrzadeh and Saravi (2010), Popescu et al. (2014), Zahmanov et al. (2015), Kaya et al. (2019), Păvăloiu et al. (2019), Motevalli-Haghi et al. (2020)
*Sambucus racemosa*	BHT EtOH Formic acid MeOH Water	Flower Fruit Leaf	Anthocyanins Flavonoids Phenols	*In vitro*	Phytocompounds enhance immune response by increasing NO, PGE2, iNOS, COX-2, IL-1β, TNF-α, and phagocytic activity. Their antioxidant capacity helps neutralize free radicals associated with human diseases	Masuda et al. (2012), Mikulic-Petkovsek et al. (2014), Todorovic et al. (2017), Choi et al. (2024)
*Vaccinium myrtillus*	Acetone EtOH MeOH Water	Flower Fruit Leaf	Anthocyanins Flavonoids Lignans Phenols Phytosterols Terpenoids	*In vivo* *In vitro*	Antidiabetic effects result from inhibition of α-amylase and α-glucosidase, complemented by antioxidant-mediated cardioprotection. Anticancer activity involves apoptosis induction and disruption of microtubule polymerization. Antimicrobial effects stem from inhibition of adhesion, cell wall disruption, and nucleic acid damage. Anti-inflammatory action attenuates cytokines (TNF-α, IL-1β, IL-6, COX-2) and LPS-induced chemokines. Additional benefits include enhanced angiogenesis for ocular health and anti-obesity effects via reduced lipid absorption, adipogenesis inhibition, increased lipolysis, and suppression of pro-inflammatory adipokines	Ferlemi and Lamari (2016), Chehri et al. (2022), Karcheva-Bahchevanska et al. (2023), Martău et al. (2023), Cvetkova et al. (2024), Mercado et al. (2025)
*Vaccinium vitis-idaea*	Acetic acid Acetone EtOH MeOH Water	Fruit Leaf Root Shoot	Anthocyanins Flavonoids; organic Acids Phenols Phytosterols Terpenoids Vitamins	*In vivo* *In vitro*	Edible organs, particularly fruits, act as prebiotics supporting gut microbiota. Phytocompounds protect cells from oxidative damage. Anticancer effects involve apoptosis and reduced proliferation, especially in estrogen receptor-positive breast cancer. Antidiabetic activity results from inhibition of α-amylase and α-glucosidase. Anticholesterolemic action stems from pancreatic lipase inhibition, reducing triglyceride absorption. Antimicrobial effects include cytotoxicity, viral replication inhibition, and prevention of microbial adhesion to epithelial cells. In oral health, extracts lower aMMP-8 levels, combating gingivitis and infections. Anti-inflammatory activity inhibits NADPH oxidase, oxidative burst, and hyaluronic acid degradation	Pop et al. (2015), Shamilov et al. (2020), Vilkickyte et al. (2022), Pärnänen et al. (2024)
*Viscum album*	EtOH Water	Flower Fruit Leaf Stem	Aminoacids Alkaloids Flavonoids Phenols Protein Sugars Terpenoids	*In vitro* *In vivo*	Anticancer activity involves inhibition of protein synthesis, ribosome inactivation, and apoptosis via selective receptor binding (e.g., galectins, L-galactono-1,4-lactone dehydrogenase). Immunomodulatory effects enhance IL-12 production through CD75 receptor targeting. Anti-inflammatory effects are driven by selective COX-2 inhibition and mRNA destabilization, reducing PGE2 levels. Phytocompounds also exhibit broad antioxidant activity against ROS and radical toxins	Hajtó et al. (2011), Song et al. (2021b), Thronicke et al. (2022), Nicoletti (2023)
*Abies alba*	Acetone EtOH Ethyl acetate MeOH; n-hexane Toluene Water	Bark Branch Cone; Knot Leaf Seed Shoot Woody parts	Lignans Phenols Terpenoids	*In vitro* *In vivo*	The extract and oil exhibit high antioxidant capacity, neutralizing ROS, glutathione oxidation, and toxins. Abies alba extract shows antimicrobial activity against fungi, bacteria, and yeast. Cytotoxic effects against breast cancer cells (>100 μg/mL) involve apoptosis and tumor suppression. Prebiotic effects support gut and vaginal microbiota. Antidiabetic action arises from inhibition of α-amylase, α-glucosidase, and DPP4. Cardioprotective effects include ACE inhibition and reduced cholesterol synthesis. Anti-psoriatic and dermatological benefits result from IL-1β suppression, reducing skin inflammation	Karkabounas et al. (2000), Ancuceanu et al. (2023)
*Aesculus hippocastanum*	Diethyl ether Ethyl acetate Methylene chloride MeOH; n-butanol n-hexane	Bark Flower Leaf Seed Stem	Coumarins Flavonoids Fatty Acids Phenols Saponins Terpenoids	*In vitro* *In vivo*	Phenolic compounds exhibit strong antioxidant activity by inhibiting ROS and oxidative damage. Extracts show effective antimicrobial activity with low MIC values against both Gram-positive and Gram-negative bacteria. Cardioprotective and anticoagulant effects result from reducing ROS, lipid peroxidation, and oxidative markers like 3-nitrotyrosine and TBARS, while supporting endogenous and non-enzymatic antioxidant systems. Dermatological protection includes shielding skin from UVB-induced DNA damage by preventing cyclobutane pyrimidine dimer formation	Jarzębski et al. (2019), Čukanović et al. (2020), Owczarek et al. (2021), Babich et al. (2024)
*Betula pendula*	EtOH MeOH Methylene chloride Water	Bark Leaf	Flavonoids Phenols Terpenoids	*In vitro* *In vivo*	Dermatological effects involve tyrosinase inhibition, reducing melanin production and dermal inflammation. Cytotoxicity reflects mitochondrial inhibition and LDH release from damaged cells. Antioxidants suppress ROS, H_2_O_2_, and microbial/influenza agents. Anticancer activity includes apoptosis, antiproliferation, and DNA fragmentation. Anti-inflammatory effects result from prostaglandin biosynthesis inhibition. Anti-arthritic action stems from xanthine oxidase inhibition and lymphocyte suppression. Anti-osteoarthritis activity involves downregulation of MMP-3, MMP-13, PGE2, and COX-2, protecting proteoglycans and collagen. Gastroprotection includes reduced lipid peroxidation and preservation of sulfhydryl groups	Rastogi et al. (2015), Ostapiuk et al. (2021)
*Castanea sativa*	Acetone EtOH Ethyl acetate MeOH NaOH Sodium sulfite Water	Bark Flower Fruit Leaf Seed	Tannins	*In vitro* *In vivo*	Antioxidants protect cells from oxidative damage, DNA injury, lipid and cholesterol oxidation, and brain stress. Antidiabetic effects involve α-glucosidase inhibition. Cardioprotection supports balanced heart contractility and vascular pressure. Antimicrobial activity inhibits microbial growth and enhances antibiotic efficacy. Anticancer effects include apoptosis, necrosis, and selective cytotoxicity. Hepatoprotection modulates TNF-α and interleukins linked to NAFLD and NASH. Anti-inflammatory effects downregulate COX-2 while modulating key cytokines. Gastroprotection involves mucosal hydration and iron chelation. Anti-aging benefits result from MMP inhibition and stimulation of type I procollagen synthesis	Živković et al. (2009b), 2009a; Jukić et al. (2014), Brizi et al. (2016), Žitek Makoter et al. (2024)
*Fagus sylvatica*	EtOH Water	Bark Leaf	Phenols Sugar and polysaccarides	*In* *vitro* *In vivo*	Endogenous antioxidants like SOD, catalase, and GPx protect against ROS. Anticancer activity inhibits tumor cell viability and metastasis. Antimicrobial effects are defined by MIC and MBC/MFC values. Antidiabetic action delays carbohydrate digestion via intestinal α-glucosidase inhibition. Antitussive effects stem from glucuronoxylan sulfates forming a protective mucosal layer, reducing cough receptor sensitivity	Sivová et al. (2016), Coşarcă et al. (2019), Tanase et al. (2021)
*Fraxinus excelsior*	MeOH Water	Bark Bud Leaf Seed	Coumarins Flavonoids Phenols Terpenoids	*In vitro* *In vivo*	Neuroprotective effects include reduced NOS/ROS, amyloid-β, and p-tau accumulation, along with increased MMPs and decreased brain glycation. Anti-inflammatory activity suppresses IL-6, IL-1β, TNF-α, and COX enzymes. Anticancer effects involve enhanced apoptosis, DNA fragmentation, and reduced tumor invasion. Antimicrobial action inhibits topoisomerases I/II and disrupts microbial synthesis. Cardioprotection is linked to improved vascular relaxation and oxidative stress reduction. Antimalarial effects arise from inflammation modulation and inhibition of *Plasmodium falciparum* asexual stages	López-Carreras et al. (2013), Sarfraz et al. (2017), Iranpanah et al. (2024)
*Fraxinus ornus*	EtOH; n-hexane	Bark Flower Leaf	Coumarins Flavonoids Phenols Phytosterols Sugars and polysaccharides	*In vitro* *In vivo*	Anti-inflammatory effects result from suppression of both classical and alternative inflammatory pathways. Methylated phenolic compounds inhibit bacterial, fungal, and viral replication. Wound healing is enhanced by accelerated epithelialization	Al-Snail (2018), Čopra-Janićijević et al. (2024)
*Juniperus communis*	Acetone EtOH MeOH Water	Fruit	Anthocyanins Flavonoids Phenols Terpenoids	*In vitro* *In vivo*	Antimicrobial effects arise from membrane rupture and inhibition of microbial proliferation and biofilm formation. Anti-hyperpigmentation and anticancer (melanoma) activity involve tyrosinase inhibition and B16F10 protein regulation. Neuroprotection is linked to reduced amyloid-β accumulation, mitigating neurodegeneration	Cioanca et al. (2014), Jegal et al. (2017), Tang et al. (2019), Čmiková et al. (2024)
*Larix decidua*	EtOH Ethyl acetate Glycerol MeOH; n-butanol n-heptane n-hexane Water	Bark Bud Leaf	Fatty Acids Flavonoids Organic Acids Phenols Sugars and polisaccharides Terpenoids	*In vitro* *In vivo*	Wound healing is enhanced via keratinocyte-driven epithelialization. Antimicrobial effects inhibit bacterial and fungal proliferation. Anticancer activity reduces tumor viability and metastasis through cytotoxicity. Dermoprotective effects involve inhibition of collagenase, elastase, and tyrosinase, preserving skin integrity. Renal and pulmonary protection involves TRPC6-mediated Ca^2+^ entry blockade. Blood purification and anti-inflammatory actions regulate stress, apoptosis, and immune-related genes. Antioxidant activity neutralizes ROS and toxic species	Salem et al. (2016), Baldan et al. (2017), Batista et al. (2022), Goels et al. (2022)
*Picea abies*	EtOH HCl HNO_3_; n-hexane Water	Bark Bud Leaf	Minerals Phenols Terpenoids Vitamins	*In vitro* *In* *vivo*	Antioxidant activity neutralizes free radicals, reducing oxidative stress. Antimicrobial effects result from membrane disruption by Picea extracts, leading to loss of viability, biofilm destruction, and impaired oxidative phosphorylation. Wound-healing is supported by enhanced keratinocyte-driven epithelialization	Rautio et al. (2007), Radulescu et al. (2011), Kreps et al. (2017), Tanase et al. (2021), Goels et al. (2022), Sandulovici et al. (2024)
*Pinus cembra*	Dichloromethane EtOH MeOH Water	Bark Fruit Leaf Seed Shoot	Anthocyanins Fatty Acids Flavonoids Phenols Terpenoids	*In vitro* *In vivo*	Antidiabetic effects result from inhibition of α-glucosidase and α-amylase. Elevated antioxidant activity, enhanced by high-altitude UV exposure, underlies dermo-cardioprotective, anti-inflammatory, and anti-rheumatic effects via reduced lipid peroxidation and oxidative stress markers. Anticancer activity involves cytotoxicity, reduced protein synthesis, and increased apoptosis	Apetrei et al. (2011), Lis et al. (2017), Nikolić et al. (2018), Chizzola and Müllner (2021), Alperth et al. (2023), Coman et al. (2023)
*Pinus mugo*	EtOH MeOH Water	Bark Bud Fruit Leaf Shoot	Terpenoids	*In vitro*	Antimicrobial activity inhibits fungal and bacterial growth. Antioxidants neutralize neutrophil-derived ROS. Anti-inflammatory and cardioprotective effects stem from inhibiting LDL oxidation, diene conjugation, and PMN activity. Wound-healing is supported by enhanced keratinocyte-driven epithelialization	Grassmann et al. (2003), Stevanovic et al. (2005), Venditti et al. (2013), Hajdari et al. (2015), Lis et al. (2019), Duțu et al. (2022)
*Pinus sylvestris*	Acetone Water	Bark Bud Fruit Leaf Shoot	Phenols	*In vitro* *In vivo*	Anti-inflammatory effects involve inhibition of COX enzymes, reducing prostaglandin E2, nitric oxide, IL-6, and MCP-1. Anticancer activity promotes apoptosis and reduces COX-2, PGE2, ERK1/2 phosphorylation, and NF-κB activation. Antimicrobial action disrupts membrane integrity, causing leakage and cell death	Karonen et al. (2004), Laavola et al. (2015), Duțu et al., 2022; Intas (2023), Macovei et al. (2023)
*Populus tremula*	Acetone EtOH MeOH Propan-2-ol Water	Bark Bud Leaf Shoot Trunk	Flavonoids Phenols	*In vitro* *In vivo*	Anticancer effects involve cytotoxicity, reduced cell viability, and inhibited tumor proliferation. Anti-inflammatory activity suppresses cytokines (IL-13, TNF-α), chemokines (IL-6, IL-8, MCP-1, Gro-α), and prostaglandins (PGE2, PGI2, PGD2). Antioxidant action blocks lipid peroxidation and neutralizes ROS and free radicals	Gundermann et al. (2020); Meyer et al. (1995), Neacsu et al. (2007), Keefover-Ring et al. (2014), Kis et al. (2021), Tsvetkov et al. (2022)
*Prunus avium*	EtOH Water	Flower Fruit Leaf Stem (Peduncle)	Anthocyanins Flavonoids Phenols Sugar and polysaccharides	*In vitro* *In vivo*	Antioxidant and anti-inflammatory effects in the gastrointestinal tract reduce MDA, ROS, and enzyme activity (NADPH oxidase, COX, NOS). Anti-obesity and antidiabetic actions involve inhibition of sugar transporters (SGLT1, GLUT2, GLUT5) and digestive enzymes. Antimicrobial activity disrupts bacterial membranes and organelles. Diuretic effects enhance renal excretion of toxins like creatinine. Cardioprotective effects improve lipid profiles, lower HbA1c and blood pressure, and reduce atherosclerotic plaques. Gastroprotection involves enhanced epithelial interaction and reduced intestinal clearance. Anticancer effects include inhibition of proliferation and promotion of apoptosis	Beconcini et al. (2020), Faienza et al. (2020), Nunes et al. (2021), 2022; Barreto-Peixoto et al. (2023)
*Prunus spinosa*	EtOH HCl MeOH Water	Flower Fruit Leaf	Fatty Acids Flavonoids Phenols Vitamins	*In vitro* *In* *vivo*	Antimicrobial effects involve membrane disruption and inhibition of vital bacterial processes. Antidiabetic and antioxidant actions lower blood glucose, reduce lipid peroxidation, and alleviate oxidative stress. Anti-inflammatory effects suppress TLR4–NF-κB signaling and cytokine production (IL-6, IRAK-1). Wound-healing and anti-aging benefits are linked to enhanced cell migration and upregulation of miR-146a	Aliyazicioglu et al. (2015), Băbălău-Fuss et al. (2021), Coppari et al. (2021), Temiz and Okumus (2022), Negrean et al. (2023)
*Pyrus pyraster*	MeOH Water	Bud Flower Fruit Leaf	Flavonoids Phenols	*In vitro* *In vivo*	Antidiabetic effects stem from α-glucosidase and α-amylase inhibition. Antimicrobial activity disrupts bacterial enzymes, protein synthesis, and DNA replication. Anticancer properties inhibit PAH-induced cell proliferation. Antioxidants reduce ROS and malondialdehyde levels. Anti-inflammatory effects lower C-reactive protein. Cardioprotection involves improved endothelial function, lipid regulation, and ACE inhibition. Urological protection counteracts cyclophosphamide-induced bladder damage. Dermatological and anti-hyperpigmentation effects result from tyrosinase inhibition	Wang et al. (2023), Elankathirselvan et al. (2024), Molinu et al. (2024), Samkaria et al. (2024)
*Quercus petraea*	EtOH NaOH; n-hexane Water	Bark Fruit Seed	Flavonoids Phenols Tannins	*In vitro* *In vivo*	Antimicrobial effects involve membrane disruption, respiratory inhibition, and DNA damage. Extracts inhibit α-glucosidase, α-amylase, α-tyrosinase, and AChE, supporting antidiabetic, neuroprotective, and skin-protective roles. Anticancer activity results from cytotoxic inhibition of tumor cell proliferation. Antioxidants neutralize ROS, contributing to anti-inflammatory, gastrointestinal, and wound-healing effects	Tumen et al. (2018), Nisca et al. (2023), Coman et al. (2024)
*Quercus pubescens*	EtOH Water	Bark	Flavonoids Phenols Tannins	*In vitro* *In vivo*	The antimicrobial action disrupts membranes, impairs respiration and DNA, leading to cell death. Extracts inhibit α-glucosidase, α-amylase, α-tyrosinase, and AChE, supporting antidiabetic, neuroprotective, and skin-protective effects. Anticancer activity arises from cytotoxic inhibition of tumor proliferation. Antioxidant effects counteract oxidative stress, underpinning related anti-inflammatory, gastrointestinal, and wound-healing properties	Nisca et al. (2023)
*Quercus robur*	Acetone EtOH Water	Bark Bud	Flavonoids Phenols Tannins Terpenoids	*In vitro*	Antioxidant activity neutralizes ROS and oxidative stress. Cynaroside, a key flavonoid, supports gastrointestinal, urological, and dermatological health. Antimicrobial effects are linked to antioxidant-driven inhibition of microbial growth	Ryabov et al. (2021), Teterovska et al. (2023)
*Salix alba*	EtOH MeOH Water	Bark Leaf	Flavonoids Phenols	*In vitro* *In vivo*	Neuroprotection occurs via AChE inhibition, while antioxidant effects combat oxidative stress. The extracts also exhibit antimicrobial activity and reduce inflammation by preserving tight junctions and downregulating NF-κB, COX-2, and TNF-α. Anti-psoriatic effects stem from modulating immune cell infiltration and keratinocyte hyperproliferation	Harbourne et al. (2009), Kenstavičienė et al. (2009), Qadir et al. (2020), Aleman et al. (2023), Gandhi et al. (2023), Sandulovici et al. (2024)
*Salix caprea*	Acetone EtOH MeOH Water	Bark Bud Flower Leaf	Flavonoids Organic acids Phenols Vitamins	*In vitro* *In vivo*	Anti-inflammatory effects result from inhibition of LPS-induced NO, COX enzymes, and prostaglandin synthesis. Antioxidant activity neutralizes ROS, while anticancer effects prevent chemically induced skin carcinogenesis. Antimicrobial action stems from antioxidant-mediated suppression of microbial growth. Neuroprotection involves modulation of catecholamine release and Ca^2+^-dependent neurotransmission	Pohjamo et al. (2003), Alam et al. (2006), Moohammadnor et al. (2010), Calorio et al. (2017), Gligorić et al. (2019b), Jang et al. (2022)
*Salix pentandra*	MeOH Resorcinol	Bark	Flavonoids Phenols	*In vitro*	Antioxidant effects occur via oxidative enzyme inhibition and Nrf2 pathway activation. Anti-inflammatory action mimics NSAID activity	Förster et al. (2021)
*Salix purpurea*	EtOH MeOH Resorcinol Water	Bark Leaf	Flavonoids Phenols	*In vitro* *In vivo.*	The antioxidant action neutralizes ROS, including superoxide and H_2_O_2_. Neuroprotection involves AChE inhibition. Compared to synthetic drugs, salicylic acid shows lower GI absorption but can damage gastric mucosa via macromolecule acetylation. Anti-inflammatory effects are mediated by inhibition of prostaglandins and COX enzymes	Pobłocka-Olech et al. (2010), Sulima et al. (2017), Gligorić et al. (2019a), Köhler et al. (2020)
*Sambucus nigra*	EtOH Water	Flower Fruit Leaf Root	Anthocyanins Flavonoids Organic acids Phenols Sugars and polysaccarides Terpenoids Vitamins	*In vitro* *In vivo*	The compounds exert strong antioxidant, anti-inflammatory, and neuroprotective effects by modulating ROS, preserving glutathione, regulating mitochondrial enzymes, and reducing pro-inflammatory cytokines. They enhance cognitive function, inhibit viral and microbial growth, and support antidiabetic and cardioprotective actions via enzyme inhibition and lipid regulation. Anticancer effects occur through apoptosis induction and signaling pathway modulation. Additional benefits include UV protection, immune stimulation, and mild diuretic action	Sidor and Gramza-Michałowska (2015), Petruţ et al. (2017), Porter and Bode (2017), Asgary and Pouramini (2022), Corrado et al. (2023), Stępień et al. (2023), Merecz-Sadowska et al. (2024)
*Sorbus aucuparia*	Chloroform Diethyl ether EtOH Ethyl acetate MeOH; n-butanol Petroleum ether Propylene glycolic Water	Flower Fruit	Anthocyanins Carotenoids Flavonoids Phenols Tocopherols Vitamins	*In vitro* *In vivo*	Nephroprotective effects preserve renal cells from oxidative damage, reducing biomarkers like KIM-1 and iNAG. Antioxidant action neutralizes ROS, including superoxide and H_2_O_2_, by modulating SOD, CAT, and GPx. Antimicrobial properties act against both Gram-positive and Gram-negative bacteria. Antidiabetic effects arise from inhibition of α-glucosidase and α-amylase. Cardioprotective activity involves thrombin and hyaluronidase inhibition and enhanced plasma antioxidant capacity. Dermoprotective effects regulate melanin synthesis via tyrosinase inhibition	Šavikin et al. (2017), Stanković et al. (2019), Rutkowska et al. (2021), 2023; Aurori et al. (2023), Aurori et al. (2024)
*Tilia cordata*	Acetone EtOH MeOH; n-hexane Water	Flower Fruit Leaf	Alkaloids Carotenoids Fatty acids Flavonoids Phenols Phytosterols Terpenoids Vitamins	*In vitro* *In vivo*	Antioxidant effects include DNA protection, reduced lipid peroxidation, cancer and heart disease risk reduction, free radical scavenging, and activation of detoxifying enzymes via the Nrf2 pathway. Anti-inflammatory activity involves modulation of TNF, NOS2, and interleukins (IL-2, IL-6, IL-8, IL-10). The antispasmodic effect results from inhibition of myosin kinase, Ca^2+^ signaling, and atropine modulation. Gastrointestinal effects are mediated by modulation of enteric neurotransmitter release affecting smooth muscle contractility	Al-Essa et al. (2007), Wilczak et al. (2013), Oniszczuk and Podgórski (2015), Cittan et al. (2018), Siger et al. (2021), Symma et al. (2021)
*Tilia platyphyllos*	Acetone EtOH; n-hexane Water	Flower Fruit Leaf	Alkaloids Carotenoids Coumarins Fatty acids Flavonoids Phenols Phytosterols Terpenoids Vitamins	*In vitro* *In vivo*	Anti-inflammatory effects arise from cytokine inhibition and suppression of mitogen-induced lymphocyte proliferation. Antioxidant activity counteracts ROS/NOS, reduces DNA damage and lipid peroxidation, and lowers cancer risk	Bardakci et al. (2020), Siger et al. (2021), Symma et al. (2021), Kędzierska et al. (2023)

### 3.4 Phytochemical composition of medicinal woody plants

The phytochemical analysis of the 54 medicinal woody species examined in this review reveals a remarkable diversity of bioactive compounds, distributed across numerous structural classes. These include phenolic acids, flavonoids, anthocyanins, tannins, lignans, alkaloids, terpenoids, saponins, fatty acids, sterols, organic acids, vitamins, and amino acids. Each taxon exhibits a unique phytochemical fingerprint that supports its traditional therapeutic applications and pharmacological profiles. Across both trees and shrubs, phenolic compounds were the most frequently reported class, present in over 80% of the species analyzed. These include hydroxycinnamic acids (e.g., chlorogenic, caffeic, ferulic), hydroxybenzoic acids (e.g., gallic, protocatechuic), and complex derivatives such as rosmarinic and ellagic acid. Phenolic acids were predominantly identified in the bark, leaves, and fruit tissues of species such as *C. monogyna* (Alirezalu et al., 2018)*, C. vulgaris* (Bekkai et al., 2022)*, R. canina* (Ayati et al., 2021), and *R. idaeus* (Krauze-Baranowska et al., 2014), supporting their antioxidant, anti-inflammatory, and cardioprotective activities (Özcandır et al., 2024).

To consolidate the chemical diversity of the species studied, the major phytochemical compounds were categorized by chemical classes. These categories serve as a framework to understand the bioactive potential and structure–activity relationships of the taxa examined. The principal compound classes identified in the woody medicinal species included in this review, based on phytochemical profiling and literature reports is reported (Table 7).

**TABLE 7:** Main phytochemical compounds present in woody species according to chemical categories.

Chemical category	Phytochemical compounds
Anthocyanins	Delphinidin-3-glucoside, cyanidin-3-glucoside, chrysanthemin, tulipanin, antirrhinin, callistephin, protoanthocyanidin A, B, A2, B1, B2, peonidin-3-glucoside, cyanidin-3-O-rutinoside, cyanidin-3-O-sambubioside, malvidin-3-O-glucoside, pelargonidin-3-glucoside, pelargonidin-3-O-rutinoside
Amino acids	Tryptophan, arginine, phenylalanine, serine, glutamic acid, aspartic acid, glycine, proline, lysine, nicotinamide, acetamide, cysteine
Alkaloids	Berberine, oxyaconthine, berbamine, brolicin, columbamine, tiliines A,B, tiliamines A,B, tilacetine A, B, N-cynnamoyl spermidine, iminodibenzoic acid
Alkanes	Pentadecane, eicosane
Carotenoids	a,β,δ – carotenes, lycopene, zeaxanthin, β-cryptoxanthin, lutein
Coumarins	Esculin, fraxin, p-coumaric acid, fraxetin, esculetin, isofraxidin, alongside, fraxidin, fraxinol, excelsides A,B, cichoriin, scopoletin, fraxin-8-β-D-glucoside
Flavonoids	Quercetin, kaempferol, rutin, isoquercetin, apigenin, luteolin, naringenin, astragalin, nicotiflorin, isorhamnetin, isoxanthohumol, xanthohumol B,C, hesperetin, homoorientin, diosmetin, eupafolin, ononin, genistein
Fatty acids	Palmitic acid, oleic acid, linoleic acid, stearic acid, palmitoleic acid, capric acid, lauric acid, myristic acid, myristoleic acid, pentadecanoic acid, linolenic acid, arachidic acid, eicosenoic acid, behenic acid, tricosanoic acid, lignoceric acid, hexadecenoic acid, margarinic acid, malvitic acid
Lignans	Metaresinol, lariciresinol, secoisolariciresinol, rubuolin A, D, rubustin E, lirioresinol A, syringaresinol, secoisolariciresinol, olivil, olivil monoacetate, icariside E, A, dihydrodehydrodiconiferyl alcohol, lyoniside
Minerals	Ca, K, Fe, Zn, Mn, Cu
Organic acids	Malic acid, tartaric acid, succinic acid, citric acid, oxalic acid, humulone, cohumulone, adhumulone, posthumulone, prehumulone, lupulone, colupulone, adlupulone, postlupulone, prelupulone, quinic acid
Phenols	Gallic acid, arbutin, catechin, epicatechin, hydroquinone, salicylic acid, pyrogallol, catechol, salvianolic acid, caffeic acid, chlorogenic acid, aesculetin, hydroxycinnamic acid, protocatechuic acid, hydrobenzoic acid, vanillic acid, caffeoylquinic acid, apigenin, vitexin, syringic acid, rosmarinic acid, neochlorogenic acid, isochlorogenic acid, gallocatechin, epigallocatechin, verbascoside, cinnamic acid
Proteins	Viscolectins, viscotoxins A1, A2, A3, B, B2, B5, B6, B7, B8, C1, 1-PS, U-PS, viscumin, mistletoelectins I, II, III, galectines
Phytosterols	β - sitosterol, α-amyrin, lupeol, ergosterol, stigmasterol, lansterol, amyrins, campesterol, squalene, tocopherols, tocotrienols, campesterols, avanasterol
Sugars and polysaccharides	Glucose, fructose, saccarose, dextrose, mannans, galactomannans, glucomannas, xyloglucans, xylands, arabinoxylan, glucuronoxylan sulfate, arabinose, galactose, galacturonic acid, sucrose, xylose
Saponins	Hederacoside B, C, D, E, F, G, H, I, α,β- hederin hederagenin, helixoside A, B, echinocystic acid derivates, ruscogen, ruscogenin, neoruscogenin, euparone, ruscogenin
Terpenoids	Limonene, α,β – pinene, camphene, α,β – phellandrene, bornyl acetate, aescin, lupeol, stigmasterol, urosolic acid, botulin, betulinic acid, thujone, germacrene B, D, cadinene, α-bergamotene, α-santalene, oleanolic acid, nerol, myrtenol, p-cymene, menthol, geraniol, abietane, ornoside, ligstroside, framoside, oleuropein, escuside, ornosol, insulahoside, hydroxyornoside, tyrosol, hydroxyframoside, β – caryophyllene, sabinene, β –elemene, furfurol, myrcene, caryophyllene oxide, humulene, linalool, terpinolene, α,γ – terpinene, terpineols, nerolidol, borneol, α-bisabolol, 1,8-cineole, cadinol, thymol, verbenone, abietic acid, abietol, larixol, citronellol
Tannins	Ellagitannins, ellagic acid, vescalagin, castalin, vescalgin, tannic acid, strictinin, isostrictinin, casuarinin, casuarictin, sanguiin H6, lambertianin C
Tocopherols	α,β,γ,δ-tocopherol
Vitamins	A, B1, B2, B3, C, D, E, K, riboflavin, folic acid

Flavonoids, including flavonols (quercetin, kaempferol), flavones (apigenin, luteolin), and isoflavones (genistein, formononetin), were highly prevalent in leaves, flowers, and fruits. For example, *E. carnea* (Veličković et al., 2017), *G. tinctoria* (Sharifi-Rad et al., 2021), and *V. myrtillus* (Chehri et al., 2022) exhibited significant quantities of flavonoid aglycones and glycosides, often linked to anti-inflammatory, cytotoxic, and hormone-modulating effects. Anthocyanins, particularly cyanidin and delphinidin derivatives, were primarily found in the fruits of species such as *R. petraeum* (Šikšnianas et al., 2013), *B. vulgaris* (Imenshahidi and Hosseinzadeh, 2019), and *Rosa pendulina* (Kunc et al., 2023b). These compounds contribute not only to pigmentation but also to notable antioxidant and anti-diabetic properties through reactive oxygen species (ROS) scavenging and enzyme inhibition (e.g., α-glucosidase) (Nazıroğlu et al., 2013). Some species, such as *R. damascena*, contain multiple flavonoids derivatives with confirmed stability under controlled storage, highlighting their value for pharmaceutical and nutraceutical formulations (Nazıroğlu et al., 2013).

Tannins, especially ellagitannins and gallotannins, were prominent in species of the *Fagaceae*, *Rosaceae*, and *Ericaceae* families (Ryabov et al., 2021; Teterovska et al., 2023). These high-molecular-weight polyphenols, detected in barks and fruits, contribute to antimicrobial, astringent, and gastroprotective actions (Coşarcă et al., 2019). Among nitrogenous compounds, alkaloids were primarily reported in *B. vulgaris*, with berberine, berbamine, and oxyacanthine as major constituents (Imenshahidi and Hosseinzadeh, 2016). These isoquinoline alkaloids display potent antimicrobial, anti-inflammatory, and cardiometabolic bioactivities, aligning with both traditional and experimental pharmacological observations.

Terpenoids-mono-, sesqui-, and triterpenes-were most commonly extracted from resinous exudates, buds, and needles of coniferous trees (e.g., *Abies alba* (Ancuceanu et al., 2023), *P. mugo* (Hajdari et al., 2015) and aromatic species such as *R. damascena* (Boskabady et al., 2011; Nayebi et al., 2017). Dominant terpenoids include α- and β-pinene, limonene, germacrene D, lupeol, and oleanolic and ursolic acids. These molecules were particularly abundant in lipophilic fractions and demonstrated wide-ranging bioactivities, including antimicrobial, anti-inflammatory, and cytotoxic properties, in both *in vitro* and *in vivo* studies (Pavlović et al., 2013; Pärnänen et al., 2024; Bouhedda et al., 2024). Saponins, particularly triterpenoid saponins such as hederacoside B and α-hederin, were predominantly reported in *H. helix* (Hooshyar et al., 2014) and *Ilex aquifolium* (Pachura et al., 2022), with roles in expectorant, antifungal, and anti-inflammatory therapies. Extraction conditions markedly influenced their yield and stability, with ultrasonic-assisted and ethanol-based extractions providing higher recoveries compared to conventional maceration.

Fatty acids, including essential polyunsaturated fatty acids (PUFAs) like linoleic, linolenic, and oleic acids, were identified in seeds and fruits of *C. avellana* (Alberti et al., 2016), *Hippophae rhamnoides* (Ma et al., 2023), and *Rosa* species (Nazıroğlu et al., 2013). Their nutritional relevance and membrane-modulating effects contribute to their application in metabolic and dermatological conditions (Ariffin and Hasham, 2020). Several species exhibited significant levels of phytosterols (e.g., β-sitosterol, campesterol, stigmasterol), particularly in the oils of *C. avellana*, *H. rhamnoides*, and *R. canina* (Demirel et al., 2016; Băbălău-Fuss et al., 2021; Manzione et al., 2024). These compounds contribute to lipid-lowering, anti-inflammatory, and skin barrier-enhancing effects. Similarly, the presence of tocopherols (vitamin E homologs) and vitamin C was frequently recorded in fruits and seeds, often in concentrations comparable to or exceeding common dietary sources (Danna et al., 2022). The presence of amino acids, proteins (e.g., viscotoxins, viscolectins), and sugar polymers (e.g., arabinogalactans, xyloglucans) in some woody taxa such as *R. canina*, *V. myrtillus*, and *V. album*, adds to their immunomodulatory and cytotoxic potential, particularly in contexts such as cancer therapy and metabolic modulation (Pop et al., 2015; Thronicke et al., 2022; Macit et al., 2023).

## 4 Discussion

### 4.1 Ethnopharmacological significance of woody species

Woody plant species in the European Alps have long served as essential components of traditional medicine, where empirical knowledge of their uses has been orally transmitted through generations (Alrhmoun et al., 2025). This ethnopharmacological heritage encompasses a wide range of therapeutic applications, with uses tailored to specific plant organs and varying in relation to cultural practices, availability of resources, and ecological distribution (Danna et al., 2022). Analysis of the woody species included in this review revealed that a diverse set of plant parts is employed for medicinal purposes, reflecting a fine-grained understanding of organ-specific phytochemical profiles. Ethnobotanical records document applications across respiratory, gastrointestinal, musculoskeletal, dermatological, metabolic, and systemic health domains. Importantly, taxa such as *P. mugo*, *Q. robur*, *R. canina*, *S. nigra*, and *V. myrtillus* are prominent not only due to their rich phytochemical profiles but also because they function dually as medicinal and nutritional agents (Grassmann et al., 2003; Pop et al., 2015; Ryabov et al., 2021; Stępień et al., 2023). This suggests potentially a broader spectrum of secondary metabolite sources among shrub taxa, potentially reflecting their adaptation to diverse ecological niches, including forest edges, montane scrub, and subalpine heathlands (Pittarello et al., 2016).

The diversity of preparations-from antioxidant-rich berry syrups to resin-based distillates used as topical and respiratory remedies-reflects a refined ethnopharmacological tradition based on empirical knowledge of extraction techniques, dosage, and seasonal phytochemical optimization (Erlund et al., 2008; Ferlemi and Lamari, 2016). This sophisticated local expertise, predating formal pharmacological frameworks, continues to be a reservoir of untapped therapeutic potential (Mattalia et al., 2023; Gerner et al., 2025). However, the prevalence of woody plants in traditional use is also partly a function of ecological dominance and accessibility, often overshadowing herbaceous taxa in local pharmacopoeias. Sustainable collection practices-such as rotational harvesting during the balsamic period-have traditionally ensured both optimal phytochemical yield and minimal ecological impact (Delfine et al., 2024), underscoring the depth of ecological knowledge embedded in these traditions.

Yet, this biocultural heritage faces significant threats. Climate change, rural depopulation, and the unregulated commercialization of herbal products now risk disrupting traditional knowledge systems and degrading both plant populations and community memory (Zerbe, 2022; Alrhmoun et al., 2025). Addressing this challenge requires a policy framework that (i) formally integrates local knowledge holders in research and conservation, (ii) ensures fair benefit-sharing as outlined in the Nagoya Protocol, and (iii) couples molecular pharmacological validation with habitat preservation (Buck and Hamilton, 2011). Such measures are critical not only for safeguarding the biodiversity in the European Alps, but also for maintaining the sociocultural resilience and pharmacological richness that these woody species represent.

### 4.2 Conservation and cultural continuity challenges

These ecological descriptors provide critical context for understanding phytochemical variability and species-specific adaptation to elevational stressors. The stratified occurrence data also inform sustainable harvesting windows and habitat-specific conservation strategies. This diversity underlines the pharmacological richness of Alpine woody flora and highlights the importance of maintaining floristic heterogeneity as a reservoir of therapeutic potential. The sustainable use of medicinal woody plants in the European Alps is imperiled by an interacting suite of ecological, socio-economic and cultural pressures that erode both population viability and the intergenerational transfer of ethnopharmacological knowledge (Schütz et al., 2006; Petrovska, 2012). Although most of the 54 taxa documented in this review are not currently red-listed, the cumulative effects of climate change, land-use reconfiguration, market-driven harvesting, and demographic change are already compromising their long-term persistence. Climatic warming of roughly 2 °C since the mid-twentieth century, combined with altered precipitation regimes and more frequent extreme events, is driving an upslope migration of thermophilous species and a contraction of subalpine-–alpine habitats (Cannone et al., 2007). For high-elevation specialists such as *L. decidua*, *R. pendulina*, and *V. vitis-idaea*, these shifts fragment populations, reduce gene flow and shorten the environmental windows in which key secondary metabolites reach balsamic maxima; the pharmacological quality of harvested organs therefore fluctuates and can decline when traditional gathering dates no longer coincide with peak phytochemical expression (Cannone et al., 2007). Concurrently, valley-bottom intensification and the abandonment of high-altitude agro-pastoral mosaics are reshaping landscape structure (Carrer et al., 2020; Zerbe, 2022). Encroaching shrubs, homogenised forest canopies, and infrastructure development diminish microsites that support under-storey medicinal shrubs and restrict safe, legal access for local collectors, thereby weakening the practical connection between communities and their botanical resources (Bhattacharya, 2025). As rural populations age and younger cohorts migrate to urban centres, oral knowledge systems that have maintained detailed harvest calendars and preparation techniques are fragmenting. Commercial phytotherapeutics-often derived from a narrow set of globally traded species-tend to displace complex local remedies, further decoupling medicinal practice from ecological context and accelerating knowledge attrition (Alrhmoun et al., 2025).

Global demand for “Alpine natural products” adds an additional layer of pressure. Industrial procurement of high-value materials such as *J. communis* berries, *V. myrtillus* fruits and *R. canina* can exceed sustainable yield, particularly where harvesting is outsourced to itinerant laborers who lack place-based stewardship norms (Petelka et al., 2020). Such extraction erodes genetic diversity, encourages illegal collection in protected areas and exacerbates habitat degradation. Despite the recognition of traditional knowledge within the Convention on Biological Diversity (CBD), practical integration into cantonal forest plans, regional land-use policies, and pharmacological research agendas remain sporadic; the resulting governance gap restricts the development of conservation strategies that are both ecologically rigorous and culturally inclusive (Petelka et al., 2022). Mitigating these threats demands a multi-scalar response. High-resolution ecological monitoring-linking remote-sensing data with community phenology networks-can detect range contractions and phenological shifts early enough to guide adaptive harvest calendars. Codifying traditional rotational or non-destructive gathering methods as best-practice guidelines and embedding them in local bylaws would align customary norms with formal regulation (Petelka et al., 2022; Alrhmoun et al., 2025). Community-based cooperatives that manage communal forest parcels for medicinal-plant production can reconcile livelihood needs with biodiversity goals while ensuring fair benefit-sharing in line with the Nagoya Protocol (Buck and Hamilton, 2011). Parallel educational initiatives, such as ethnobotanical gardens and apprenticeship schemes, are indispensable for revitalizing dialect terminology, harvest rites and artisanal preparation skills. Finally, regional climate-adaptation and rural-development plans should explicitly recognize ethnopharmacological resources as both biodiversity assets and cultural ecosystem services, thereby anchoring their conservation within broader policy frameworks (Cannone et al., 2007).

Protecting Alpine medicinal woody plants is therefore not merely a matter of conserving species lists. It requires sustaining the dynamic socio-ecological relationships that bind mountain communities to their phytotherapeutic heritage. Only an integrative approach-combining phenological science, adaptive management, equitable governance and cultural revitalization-can secure the twin legacies of biological diversity and ethnopharmacological knowledge for future generations.

### 4.3 Bioprospecting and future pharmacological potential

The extreme ecological conditions of the Alpine arc-high-energy ultraviolet flux, steep thermal gradients, and widespread oligotrophic soils-select for woody plants that synthesise an unusually broad repertoire of defensive metabolites (Billings and Mooney, 1968). These compounds, which include flavonoids, stilbenes, anthocyanins, condensed tannins, clerodane and lupane triterpenes, lignans, isoquinoline alkaloids and volatile mono- and sesquiterpenes, express bioactivities that map directly onto several pressing pharmaceutical needs: chronic inflammation, insulin resistance, endothelial dysfunction, tumour progression, viral infection and multi-drug bacterial resistance (Petrovska, 2012; Petelka et al., 2020). In the present synthesis, extracts from more than two-thirds of the 54 taxa displayed two or more mechanistic endpoints *in vitro* or *in vivo*, confirming the multi-target character that modern drug-discovery programs increasingly seek (Hajipour et al., 2022).

Among the most compelling leads, berry or hip extracts combine high anthocyanin density with pronounced α-glucosidase inhibition and endothelial-protective effects, positioning them as templates for nutraceuticals targeting early-stage metabolic syndrome (Erlund et al., 2008; Buyukokuroglu and Gulcin, 2009; Ferlemi and Lamari, 2016; Ermolaev and Fedorov, 2022; Tahir et al., 2023). Ethanolic fractions of *G. tinctoria* and *E. carnea* deliver sub-micromolar cytotoxicity against breast and cervical carcinoma lines, mediated by genistein derivatives and rosmarinic-acid-rich polyphenol pools, while saponin-enriched extracts of *H. helix* exhibit synergistic antiviral and expectorant actions that merit further exploration for respiratory-tract infections (Rigano et al., 2009; Veličković et al., 2017; Al-Snafi, 2018). Less-studied taxa, notably *Myricaria germanica* and *O. spinosa*, furnish lignan and isoflavonoid scaffolds with anti-arthritic and diuretic properties, whereas the resinous tissues of *P. mugo* and *L. decidua* concentrate bornyl esters and abietane diterpenes whose potent antibacterial and dermatological activities have only recently been quantified (Grassmann et al., 2003; Benedec et al., 2012; Nawwar et al., 2013; Baldan et al., 2017). Despite these promising signals, more than 90% of the bioactivity reports remain at the pre-clinical stage; pharmacokinetic data are fragmentary, and systematic toxicity profiling is scarce. Matrix effects and compound instability-especially in polyphenol-rich fractions-pose additional obstacles to formulation.

Progressing Alpine woody metabolites from bench to bedside therefore demands an integrated platform that couples high-resolution metabolomics, network pharmacology and *in silico* docking with iterative bioassay-guided fractionation and rigorous ADME/Tox evaluation (Javid et al., 2024). Standardized extraction and reference-material repositories will be essential to ensure inter-laboratory reproducibility, while scalable green-chemistry protocols can minimize ecological footprint. Equally critical are robust access-and-benefit frameworks: most candidate species are embedded in local healing systems, and any commercial valorization must guarantee fair compensation and continued stewardship by Alpine communities. If these scientific and ethical requisites are met, the woody flora of the European Alps could yield a new generation of multi-functional agents that bridge the gap between traditional phytotherapy and precision pharmacology.

## 5 Conclusion

This systematic review highlights the untapped pharmacological and ecological potential of woody plant species in the European Alps. These taxa form an integral part of biocultural landscapes, where medicinal relevance is deeply embedded in traditional ecological knowledge and shaped by centuries of local practice. Their multifunctionality, spanning therapeutic use, ecosystem regulation, and cultural identity, demands an approach that integrates pharmacological exploration with environmental stewardship. However, this reservoir of biodiversity faces mounting pressures. Climatic shifts, habitat homogenization, unsustainable harvesting, and the erosion of intergenerational knowledge collectively endanger both the biological integrity of key species and the continuity of Alpine ethnopharmacological. At the same time, significant methodological limitations-ranging from inconsistent extraction techniques and bioassay designs to insufficient toxicological profiling-hinder the reproducibility and translational potential of existing findings. In this context, our synthesis underscores the need to simultaneously advance scientific validation and conservation-oriented management of these taxa. Future research must be interdisciplinary, coupling advanced metabolomics and target deconvolution with ecologically grounded fieldwork and participatory ethnobotany. Standardized protocols and taxonomic verification are essential to ensure scientific reliability, while equitable frameworks, such as those outlined in the Nagoya Protocol, must guide benefit-sharing and the inclusion of traditional calendars in conservation planning. By emphasizing the interdependence between pharmacological promise, ecosystem resilience, and cultural continuity, this review provides a roadmap for harnessing the therapeutic value of Alpine woody flora while safeguarding the socio-ecological systems that sustain them.

## Data Availability

The original contributions presented in the study are included in the article/Supplementary Material, further inquiries can be directed to the corresponding author.
