# Supplementary material for: Ethnopharmacology and ecosystem applications of woody plant species in the Southern European Alps: a systematic review
Source: Front Pharmacol. 2026 Mar 10;17:1729802. doi: 10.3389/fphar.2026.1729802 (PMC13008899; doi:10.3389/fphar.2026.1729802)
Supplement: Supplementary file 1 [file Table1.docx]

**Supporting Information**

**May 2025**

**Pub med: 53**

|  | **Search terms** | **Hits** |
| --- | --- | --- |
| **#1** | "Ethnopharmacology"[Title/Abstract] OR "Ethnomedicine"[Title/Abstract] OR "Medicinal ethnobotany"[Title/Abstract] OR "Ethnopharmacy"[Title/Abstract] OR "Medicine"[Title/Abstract] OR "Folk pharmacology"[Title/Abstract] OR "Pharmacology"[Title/Abstract] OR "Medical ethnobotany"[Title/Abstract] OR "Ethnotherapeutics"[Title/Abstract] OR "Phytotherapeutics"[Title/Abstract] OR "Natural-product pharmacology"[Title/Abstract] OR "Traditional phytotherapy"[Title/Abstract] OR "Traditional medicine"[Title/Abstract] OR "Herbal pharmacology"[Title/Abstract] OR "Folk medicine"[Title/Abstract] OR "Medicinal plant knowledge"[Title/Abstract] OR "Ethnopharmacognosy"[Title/Abstract] OR "Indigenous therapeutics"[Title/Abstract] OR "Medicinal phytochemistry"[Title/Abstract] OR "Ethnophytotherapy"[Title/Abstract] OR "Anthocyanins"[Title/Abstract] OR "Amino Acids"[Title/Abstract] OR "Alkaloids"[Title/Abstract] OR "Alkanes"[Title/Abstract] OR "Carotenoids"[Title/Abstract] OR "Coumarins"[Title/Abstract] OR "Flavonoids"[Title/Abstract] OR "Fatty Acids"[Title/Abstract] OR "Lignans"[Title/Abstract] OR "Minerals"[Title/Abstract] OR "Organic acids"[Title/Abstract] OR "Phenols"[Title/Abstract] OR "Proteins"[Title/Abstract] OR "Phytosterols"[Title/Abstract] OR "Sugars"[Title/Abstract] OR "Polysaccharides"[Title/Abstract] OR "Saponins"[Title/Abstract] OR "Terpenoids"[Title/Abstract] OR "Tannins"[Title/Abstract] OR "Tocopherols"[Title/Abstract] OR "Vitamins"[Title/Abstract] | **2,718,887** |
| **#2** | "Woody taxa"[Title/Abstract] OR "Ligneous species"[Title/Abstract] OR "Arboreal species"[Title/Abstract] OR "Lignophytes"[Title/Abstract] OR "Phanerophytes"[Title/Abstract] OR "Fruticose species"[Title/Abstract] OR "Woody perennials"[Title/Abstract] OR "Shrubs"[Title/Abstract] OR "Trees"[Title/Abstract] OR "Woody angiosperms"[Title/Abstract] OR "Gymnosperm taxa"[Title/Abstract] OR "Woody plant species"[Title/Abstract] OR "Woody species"[Title/Abstract] OR "Woody plants"[Title/Abstract] OR "Woody vegetation"[Title/Abstract] OR "Forest flora"[Title/Abstract] OR "Woody flora"[Title/Abstract] OR "Woody dicotyledons"[Title/Abstract] OR "Shrubby vegetation"[Title/Abstract] OR "Timber species"[Title/Abstract] OR "Woody gymnosperms"[Title/Abstract] OR "Woody trees"[Title/Abstract] OR "Chamaephytes"[Title/Abstract] OR "Adoxaceae"[Title/Abstract] OR "Aquifoliaceae"[Title/Abstract] OR "Araliaceae"[Title/Abstract] OR "Asparagaceae"[Title/Abstract] OR "Berberidaceae"[Title/Abstract] OR "Betulaceae"[Title/Abstract] OR "Cannabaceae"[Title/Abstract] OR "Cupressaceae"[Title/Abstract] OR "Elaeagnaceae"[Title/Abstract] OR "Ericaceae"[Title/Abstract] OR "Fabaceae"[Title/Abstract] OR "Fagaceae"[Title/Abstract] OR "Grossulariaceae"[Title/Abstract] OR "Hippocastanaceae"[Title/Abstract] OR "Malvaceae"[Title/Abstract] OR "Oleaceae"[Title/Abstract] OR "Pinaceae"[Title/Abstract] OR "Rosaceae"[Title/Abstract] OR "Santalaceae"[Title/Abstract] OR "Salicaceae"[Title/Abstract] OR "Tamaricaceae"[Title/Abstract] OR "Arctostaphylos uva-ursi"[Title/Abstract] OR "Berberis vulgaris"[Title/Abstract] OR "Calluna vulgaris"[Title/Abstract] OR "Corylus avellana"[Title/Abstract] OR "Crataegus monogyna"[Title/Abstract] OR "Genista tinctoria"[Title/Abstract] OR "Hedera helix"[Title/Abstract] OR "Humulus lupulus"[Title/Abstract] OR "Ilex aquifolium"[Title/Abstract] OR "Myricaria germanica"[Title/Abstract] OR "Ononis spinosa"[Title/Abstract] OR "Rosa canina"[Title/Abstract] OR "Rosa corymbifera"[Title/Abstract] OR "Rosa x damascena"[Title/Abstract] OR "Rosa pendulina"[Title/Abstract] OR "Ruscus aculeatus"[Title/Abstract] OR "Sambucus ebulus"[Title/Abstract] OR "Sambucus nigra"[Title/Abstract] OR "Sambucus racemosa"[Title/Abstract] OR "Sorbus aucuparia"[Title/Abstract] OR "Vaccinium myrtillus"[Title/Abstract] OR "Vaccinium vitis-idaea"[Title/Abstract] OR "Viscum album"[Title/Abstract] OR "Abies alba"[Title/Abstract] OR "Aesculus hippocastanum"[Title/Abstract] OR "Betula pendula"[Title/Abstract] OR "Castanea sativa"[Title/Abstract] OR "Fagus sylvatica"[Title/Abstract] OR "Fraxinus excelsior"[Title/Abstract] OR "Fraxinus ornus"[Title/Abstract] OR "Juniperus communis"[Title/Abstract] OR "Larix decidua"[Title/Abstract] OR "Picea abies"[Title/Abstract] OR "Pinus cembra"[Title/Abstract] OR "Pinus mugo"[Title/Abstract] OR "Pinus sylvestris"[Title/Abstract] OR "Populus tremula"[Title/Abstract] OR "Prunus avium"[Title/Abstract] OR "Prunus spinosa"[Title/Abstract] OR "Pyrus pyraster"[Title/Abstract] OR "Quercus petraea"[Title/Abstract] OR "Quercus pubescens"[Title/Abstract] OR "Quercus robur"[Title/Abstract] OR "Salix alba"[Title/Abstract] OR "Salix caprea"[Title/Abstract] OR "Salix pentandra"[Title/Abstract] OR "Salix purpurea"[Title/Abstract] OR "Tilia cordata"[Title/Abstract] OR "Tilia platyphyllos"[Title/Abstract] | **97,897** |
| **#3** | "Alps"[Title/Abstract] OR "European Alps"[Title/Abstract] OR "Alpine region"[Title/Abstract] OR "Alpine arc"[Title/Abstract] OR "Alpine chain"[Title/Abstract] OR "Alpine orogen"[Title/Abstract] OR "Central European Alps"[Title/Abstract] OR "Western Alps"[Title/Abstract] OR "Eastern Alps"[Title/Abstract] OR "Northern Limestone Alps"[Title/Abstract] OR "Southern Limestone Alps"[Title/Abstract] OR "Alpine ecoregion"[Title/Abstract] OR "European Alpine region"[Title/Abstract] OR "Alpine"[Title/Abstract] OR "Alpine environment"[Title/Abstract] OR "Alpine range"[Title/Abstract] OR "Inner Alps"[Title/Abstract] OR "Pre-Alpine region"[Title/Abstract] | **12,965** |
| **#4** | #1 AND #2 AND #3 | **53** |

**Scopus: 801**

|  | **Search terms** | **Hits** |
| --- | --- | --- |
| #1 | TITLE-ABS-KEY ( "Ethnopharmacology" OR "Ethnomedicine" OR "Medicinal ethnobotany" OR "Ethnopharmacy" OR "Medicine" OR "Folk pharmacology" OR "Pharmacology" OR "Medical ethnobotany" OR "Ethnotherapeutics" OR "Phytotherapeutics" OR "Natural-product pharmacology" OR "Traditional phytotherapy" OR "Traditional medicine" OR "Herbal pharmacology" OR "Folk medicine" OR "Medicinal plant knowledge" OR "Ethnopharmacognosy" OR "Indigenous therapeutics" OR "Medicinal phytochemistry" OR "Ethnophytotherapy" OR "Anthocyanins" OR "Amino Acids" OR "Alkaloids" OR "Alkanes" OR "Carotenoids" OR "Coumarins" OR "Flavonoids" OR "Fatty Acids" OR "Lignans" OR "Minerals" OR "Organic acids" OR "Phenols" OR "Proteins" OR "Phytosterols" OR "Sugars" OR "Polysaccharides" OR "Saponins" OR "Terpenoids" OR "Tannins" OR "Tocopherols" OR "Vitamins" ) | **13,059,569** |
| #2 | TITLE-ABS-KEY ( "Woody taxa" OR "Ligneous species" OR "Arboreal species" OR "Lignophytes" OR "Phanerophytes" OR "Fruticose species" OR "Woody perennials" OR "Shrubs" OR "Trees" OR "Woody angiosperms" OR "Gymnosperm taxa" OR "Woody plant species" OR "Woody species" OR "Woody plants" OR "Woody vegetation" OR "Forest flora" OR "Woody flora" OR "Woody dicotyledons" OR "Shrubby vegetation" OR "Timber species" OR "Woody gymnosperms" OR "Woody trees" OR "Chamaephytes" OR "Adoxaceae" OR "Aquifoliaceae" OR "Araliaceae" OR "Asparagaceae" OR "Berberidaceae" OR "Betulaceae" OR "Cannabaceae" OR "Cupressaceae" OR "Elaeagnaceae" OR "Ericaceae" OR "Fabaceae" OR "Fagaceae" OR "Grossulariaceae" OR "Hippocastanaceae" OR "Malvaceae" OR "Oleaceae" OR "Pinaceae" OR "Rosaceae" OR "Santalaceae" OR "Salicaceae" OR "Tamaricaceae" OR "Arctostaphylos uva-ursi" OR "Berberis vulgaris" OR "Calluna vulgaris" OR "Corylus avellana" OR "Crataegus monogyna" OR "Genista tinctoria" OR "Hedera helix" OR "Humulus lupulus" OR "Ilex aquifolium" OR "Myricaria germanica" OR "Ononis spinosa" OR "Rosa canina" OR "Rosa corymbifera" OR "Rosa x damascena" OR "Rosa pendulina" OR "Ruscus aculeatus" OR "Sambucus ebulus" OR "Sambucus nigra" OR "Sambucus racemosa" OR "Sorbus aucuparia" OR "Vaccinium myrtillus" OR "Vaccinium vitis-idaea" OR "Viscum album" OR "Abies alba" OR "Aesculus hippocastanum" OR "Betula pendula" OR "Castanea sativa" OR "Fagus sylvatica" OR "Fraxinus excelsior" OR "Fraxinus ornus" OR "Juniperus communis" OR "Larix decidua" OR "Picea abies" OR "Pinus cembra" OR "Pinus mugo" OR "Pinus sylvestris" OR "Populus tremula" OR "Prunus avium" OR "Prunus spinosa" OR "Pyrus pyraster" OR "Quercus petraea" OR "Quercus pubescens" OR "Quercus robur" OR "Salix alba" OR "Salix caprea" OR "Salix pentandra" OR "Salix purpurea" OR "Tilia cordata" OR "Tilia platyphyllos" ) | **1,228,150** |
| #3 | TITLE-ABS-KEY ( "Alps" OR "European Alps" OR "Alpine region" OR "Alpine arc" OR "Alpine chain" OR "Alpine orogen" OR "Central European Alps" OR "Western Alps" OR "Eastern Alps" OR "Northern Limestone Alps" OR "Southern Limestone Alps" OR "Alpine ecoregion" OR "European Alpine region" OR "Alpine" OR "Alpine environment" OR "Alpine range" OR "Inner Alps" OR "Pre-Alpine region" ) | **133,212** |
| #4 | #1 AND #2 AND #3 | **801** |

**Web of science: 136**

|  | **Search terms** | **Hits** |
| --- | --- | --- |
| #1 | **TI=** ( "Ethnopharmacology" OR "Ethnomedicine" OR "Medicinal ethnobotany" OR "Ethnopharmacy" OR "Medicine" OR "Folk pharmacology" OR "Pharmacology" OR "Medical ethnobotany" OR "Ethnotherapeutics" OR "Phytotherapeutics" OR "Natural-product pharmacology" OR "Traditional phytotherapy" OR "Traditional medicine" OR "Herbal pharmacology" OR "Folk medicine" OR "Medicinal plant knowledge" OR "Ethnopharmacognosy" OR "Indigenous therapeutics" OR "Medicinal phytochemistry" OR "Ethnophytotherapy" OR "Anthocyanins" OR "Amino Acids" OR "Alkaloids" OR "Alkanes" OR "Carotenoids" OR "Coumarins" OR "Flavonoids" OR "Fatty Acids" OR "Lignans" OR "Minerals" OR "Organic acids" OR "Phenols" OR "Proteins" OR "Phytosterols" OR "Sugars" OR "Polysaccharides" OR "Saponins" OR "Terpenoids" OR "Tannins" OR "Tocopherols" OR "Vitamins" ) | **896,670** |
| #2 | **AB=** ( "Ethnopharmacology" OR "Ethnomedicine" OR "Medicinal ethnobotany" OR "Ethnopharmacy" OR "Medicine" OR "Folk pharmacology" OR "Pharmacology" OR "Medical ethnobotany" OR "Ethnotherapeutics" OR "Phytotherapeutics" OR "Natural-product pharmacology" OR "Traditional phytotherapy" OR "Traditional medicine" OR "Herbal pharmacology" OR "Folk medicine" OR "Medicinal plant knowledge" OR "Ethnopharmacognosy" OR "Indigenous therapeutics" OR "Medicinal phytochemistry" OR "Ethnophytotherapy" OR "Anthocyanins" OR "Amino Acids" OR "Alkaloids" OR "Alkanes" OR "Carotenoids" OR "Coumarins" OR "Flavonoids" OR "Fatty Acids" OR "Lignans" OR "Minerals" OR "Organic acids" OR "Phenols" OR "Proteins" OR "Phytosterols" OR "Sugars" OR "Polysaccharides" OR "Saponins" OR "Terpenoids" OR "Tannins" OR "Tocopherols" OR "Vitamins" ) | **3,008,303** |
| #3 | **AK=** ( "Ethnopharmacology" OR "Ethnomedicine" OR "Medicinal ethnobotany" OR "Ethnopharmacy" OR "Medicine" OR "Folk pharmacology" OR "Pharmacology" OR "Medical ethnobotany" OR "Ethnotherapeutics" OR "Phytotherapeutics" OR "Natural-product pharmacology" OR "Traditional phytotherapy" OR "Traditional medicine" OR "Herbal pharmacology" OR "Folk medicine" OR "Medicinal plant knowledge" OR "Ethnopharmacognosy" OR "Indigenous therapeutics" OR "Medicinal phytochemistry" OR "Ethnophytotherapy" OR "Anthocyanins" OR "Amino Acids" OR "Alkaloids" OR "Alkanes" OR "Carotenoids" OR "Coumarins" OR "Flavonoids" OR "Fatty Acids" OR "Lignans" OR "Minerals" OR "Organic acids" OR "Phenols" OR "Proteins" OR "Phytosterols" OR "Sugars" OR "Polysaccharides" OR "Saponins" OR "Terpenoids" OR "Tannins" OR "Tocopherols" OR "Vitamins" ) | **517,898** |
| #4 | **TI=** ( "Woody taxa" OR "Ligneous species" OR "Arboreal species" OR "Lignophytes" OR "Phanerophytes" OR "Fruticose species" OR "Woody perennials" OR "Shrubs" OR "Trees" OR "Woody angiosperms" OR "Gymnosperm taxa" OR "Woody plant species" OR "Woody species" OR "Woody plants" OR "Woody vegetation" OR "Forest flora" OR "Woody flora" OR "Woody dicotyledons" OR "Shrubby vegetation" OR "Timber species" OR "Woody gymnosperms" OR "Woody trees" OR "Chamaephytes" OR "Adoxaceae" OR "Aquifoliaceae" OR "Araliaceae" OR "Asparagaceae" OR "Berberidaceae" OR "Betulaceae" OR "Cannabaceae" OR "Cupressaceae" OR "Elaeagnaceae" OR "Ericaceae" OR "Fabaceae" OR "Fagaceae" OR "Grossulariaceae" OR "Hippocastanaceae" OR "Malvaceae" OR "Oleaceae" OR "Pinaceae" OR "Rosaceae" OR "Santalaceae" OR "Salicaceae" OR "Tamaricaceae" OR "Arctostaphylos uva-ursi" OR "Berberis vulgaris" OR "Calluna vulgaris" OR "Corylus avellana" OR "Crataegus monogyna" OR "Genista tinctoria" OR "Hedera helix" OR "Humulus lupulus" OR "Ilex aquifolium" OR "Myricaria germanica" OR "Ononis spinosa" OR "Rosa canina" OR "Rosa corymbifera" OR "Rosa x damascena" OR "Rosa pendulina" OR "Ruscus aculeatus" OR "Sambucus ebulus" OR "Sambucus nigra" OR "Sambucus racemosa" OR "Sorbus aucuparia" OR "Vaccinium myrtillus" OR "Vaccinium vitis-idaea" OR "Viscum album" OR "Abies alba" OR "Aesculus hippocastanum" OR "Betula pendula" OR "Castanea sativa" OR "Fagus sylvatica" OR "Fraxinus excelsior" OR "Fraxinus ornus" OR "Juniperus communis" OR "Larix decidua" OR "Picea abies" OR "Pinus cembra" OR "Pinus mugo" OR "Pinus sylvestris" OR "Populus tremula" OR "Prunus avium" OR "Prunus spinosa" OR "Pyrus pyraster" OR "Quercus petraea" OR "Quercus pubescens" OR "Quercus robur" OR "Salix alba" OR "Salix caprea" OR "Salix pentandra" OR "Salix purpurea" OR "Tilia cordata" OR "Tilia platyphyllos" ) | **108,996** |
| #5 | **AB=** ( "Woody taxa" OR "Ligneous species" OR "Arboreal species" OR "Lignophytes" OR "Phanerophytes" OR "Fruticose species" OR "Woody perennials" OR "Shrubs" OR "Trees" OR "Woody angiosperms" OR "Gymnosperm taxa" OR "Woody plant species" OR "Woody species" OR "Woody plants" OR "Woody vegetation" OR "Forest flora" OR "Woody flora" OR "Woody dicotyledons" OR "Shrubby vegetation" OR "Timber species" OR "Woody gymnosperms" OR "Woody trees" OR "Chamaephytes" OR "Adoxaceae" OR "Aquifoliaceae" OR "Araliaceae" OR "Asparagaceae" OR "Berberidaceae" OR "Betulaceae" OR "Cannabaceae" OR "Cupressaceae" OR "Elaeagnaceae" OR "Ericaceae" OR "Fabaceae" OR "Fagaceae" OR "Grossulariaceae" OR "Hippocastanaceae" OR "Malvaceae" OR "Oleaceae" OR "Pinaceae" OR "Rosaceae" OR "Santalaceae" OR "Salicaceae" OR "Tamaricaceae" OR "Arctostaphylos uva-ursi" OR "Berberis vulgaris" OR "Calluna vulgaris" OR "Corylus avellana" OR "Crataegus monogyna" OR "Genista tinctoria" OR "Hedera helix" OR "Humulus lupulus" OR "Ilex aquifolium" OR "Myricaria germanica" OR "Ononis spinosa" OR "Rosa canina" OR "Rosa corymbifera" OR "Rosa x damascena" OR "Rosa pendulina" OR "Ruscus aculeatus" OR "Sambucus ebulus" OR "Sambucus nigra" OR "Sambucus racemosa" OR "Sorbus aucuparia" OR "Vaccinium myrtillus" OR "Vaccinium vitis-idaea" OR "Viscum album" OR "Abies alba" OR "Aesculus hippocastanum" OR "Betula pendula" OR "Castanea sativa" OR "Fagus sylvatica" OR "Fraxinus excelsior" OR "Fraxinus ornus" OR "Juniperus communis" OR "Larix decidua" OR "Picea abies" OR "Pinus cembra" OR "Pinus mugo" OR "Pinus sylvestris" OR "Populus tremula" OR "Prunus avium" OR "Prunus spinosa" OR "Pyrus pyraster" OR "Quercus petraea" OR "Quercus pubescens" OR "Quercus robur" OR "Salix alba" OR "Salix caprea" OR "Salix pentandra" OR "Salix purpurea" OR "Tilia cordata" OR "Tilia platyphyllos" ) | **363,245** |
| #6 | **AK=** ( "Woody taxa" OR "Ligneous species" OR "Arboreal species" OR "Lignophytes" OR "Phanerophytes" OR "Fruticose species" OR "Woody perennials" OR "Shrubs" OR "Trees" OR "Woody angiosperms" OR "Gymnosperm taxa" OR "Woody plant species" OR "Woody species" OR "Woody plants" OR "Woody vegetation" OR "Forest flora" OR "Woody flora" OR "Woody dicotyledons" OR "Shrubby vegetation" OR "Timber species" OR "Woody gymnosperms" OR "Woody trees" OR "Chamaephytes" OR "Adoxaceae" OR "Aquifoliaceae" OR "Araliaceae" OR "Asparagaceae" OR "Berberidaceae" OR "Betulaceae" OR "Cannabaceae" OR "Cupressaceae" OR "Elaeagnaceae" OR "Ericaceae" OR "Fabaceae" OR "Fagaceae" OR "Grossulariaceae" OR "Hippocastanaceae" OR "Malvaceae" OR "Oleaceae" OR "Pinaceae" OR "Rosaceae" OR "Santalaceae" OR "Salicaceae" OR "Tamaricaceae" OR "Arctostaphylos uva-ursi" OR "Berberis vulgaris" OR "Calluna vulgaris" OR "Corylus avellana" OR "Crataegus monogyna" OR "Genista tinctoria" OR "Hedera helix" OR "Humulus lupulus" OR "Ilex aquifolium" OR "Myricaria germanica" OR "Ononis spinosa" OR "Rosa canina" OR "Rosa corymbifera" OR "Rosa x damascena" OR "Rosa pendulina" OR "Ruscus aculeatus" OR "Sambucus ebulus" OR "Sambucus nigra" OR "Sambucus racemosa" OR "Sorbus aucuparia" OR "Vaccinium myrtillus" OR "Vaccinium vitis-idaea" OR "Viscum album" OR "Abies alba" OR "Aesculus hippocastanum" OR "Betula pendula" OR "Castanea sativa" OR "Fagus sylvatica" OR "Fraxinus excelsior" OR "Fraxinus ornus" OR "Juniperus communis" OR "Larix decidua" OR "Picea abies" OR "Pinus cembra" OR "Pinus mugo" OR "Pinus sylvestris" OR "Populus tremula" OR "Prunus avium" OR "Prunus spinosa" OR "Pyrus pyraster" OR "Quercus petraea" OR "Quercus pubescens" OR "Quercus robur" OR "Salix alba" OR "Salix caprea" OR "Salix pentandra" OR "Salix purpurea" OR "Tilia cordata" OR "Tilia platyphyllos" ) | **60,281** |
| #7 | **TI=** ( "Alps" OR "European Alps" OR "Alpine region" OR "Alpine arc" OR "Alpine chain" OR "Alpine orogen" OR "Central European Alps" OR "Western Alps" OR "Eastern Alps" OR "Northern Limestone Alps" OR "Southern Limestone Alps" OR "Alpine ecoregion" OR "European Alpine region" OR "Alpine" OR "Alpine environment" OR "Alpine range" OR "Inner Alps" OR "Pre-Alpine region" ) | **33,548** |
| #8 | **AB=** ( "Alps" OR "European Alps" OR "Alpine region" OR "Alpine arc" OR "Alpine chain" OR "Alpine orogen" OR "Central European Alps" OR "Western Alps" OR "Eastern Alps" OR "Northern Limestone Alps" OR "Southern Limestone Alps" OR "Alpine ecoregion" OR "European Alpine region" OR "Alpine" OR "Alpine environment" OR "Alpine range" OR "Inner Alps" OR "Pre-Alpine region" ) | **58,583** |
| #9 | AK**=** ( "Alps" OR "European Alps" OR "Alpine region" OR "Alpine arc" OR "Alpine chain" OR "Alpine orogen" OR "Central European Alps" OR "Western Alps" OR "Eastern Alps" OR "Northern Limestone Alps" OR "Southern Limestone Alps" OR "Alpine ecoregion" OR "European Alpine region" OR "Alpine" OR "Alpine environment" OR "Alpine range" OR "Inner Alps" OR "Pre-Alpine region" ) | **15,908** |
| #10 | #1 OR #2 OR #3 | **3,646,881** |
| #11 | #4 OR #5 OR #6 | **424,410** |
| #12 | #7 OR #8 OR #9 | **72,006** |
| #13 | #10 AND #11 AND #12 | **133** |

Total references: 987

Unrepeated references: 807

Relevant references: 281
